# A systematic review of relationships and sex education outcomes for students with intellectual disability reported in the international literature

**DOI:** 10.1111/jir.12952

**Published:** 2022-06-13

**Authors:** L. Paulauskaite, C. Rivas, A. Paris, V. Totsika

**Affiliations:** ^1^ Social Research Institute University College London London UK; ^2^ Centre for Educational Development Appraisal and Research University of Warwick Coventry UK; ^3^ Division of Psychiatry University College London London UK; ^4^ Department of Psychiatry Monash University Melbourne Victoria Australia; ^5^ Tavistock and Portman NHS Foundation Trust London UK

**Keywords:** Core Outcome Set, intellectual disability, relationships and sex education

## Abstract

**Background:**

Little is known about how to evaluate relationships and sex education (RSE) delivered to students with intellectual disability and what stakeholders perceive are important outcomes. The present study aimed to systematically review existing studies on outcomes of RSE, as the first step in the development of a core outcome set (COS) for students with intellectual disability.

**Method:**

A systematic literature process included two stages: (1) searching for studies reporting on RSE outcomes for students with intellectual disability and (2) studies reporting on measurement properties (e.g. validity, reliability and responsiveness) of standardised instruments identified in stage 1.

**Results:**

A total of 135 RSE outcomes were extracted from 42 studies: 43 outcomes for students in secondary education and 92 outcomes for students in further education. No RSE outcomes were reported for primary education. Outcomes referred to the human body, hygiene, relationships, sexuality, sex and its consequences, inappropriate and appropriate social and sexual behaviour, keeping safe, emotional vocabulary and positive self‐esteem. Outcomes were predominantly knowledge‐based, rather than relating to skills and attitudes development. Students with intellectual disability, parents and teachers perceive different RSE outcomes meaningful. Five instruments were used to measure the outcomes, but none have established psychometric properties with this population.

**Conclusions:**

The comprehensive list of RSE outcomes for students with intellectual disability will be used to inform the next steps of a Core Outcome Set needed for RSE evaluations in research and education settings. There is an urgent need to develop standardised instruments validated for students with intellectual disability.

## Introduction

Current evidence suggests that individuals with intellectual disability have limited knowledge and skills regarding sexuality and relationships (Galea *et al*. 2004; Jahoda and Pownall 2014; Borawska‐Charko *et al*. 2017; Baines *et al*. 2018). They are twice as likely to experience unintended pregnancies, sexually transmitted diseases, and sexual abuse than people in the general population (Jahoda and Pownall 2014; Baines *et al*. 2018).

In 2020, relationships and sex education (RSE) became compulsory in English schools for all students including those with intellectual disability (Department for Education [DfE] 2019). The aim of RSE is to equip all students with knowledge, attitudes, and skills about ‘the emotional, social and physical aspects of growing up, relationships, sex, human sexuality and sexual health’ (Sex Education Forum 2020). The new policy indicates that all students including those with intellectual disability must receive Relationships Education in primary schools (when students are 5–11 years) and RSE in secondary schools (when students are 11–16 years) (DfE 2019). Schools can adapt the content to meet individual needs and developmental levels and RSE frequency, duration, and mode of delivery is not specified (DfE 2019). Across the UK, there is lack of standardisation of RSE delivery and content (Lafferty *et al*. 2012). For example, RSE is not compulsory in Scotland whereas in Northern Ireland this education is compulsory (although parents have a right to withdraw their children from parts of this education in primary schools and schools can develop their own content of RSE lessons to match the schools' ethos) and from 2022 RSE became compulsory in Wales (The Scottish Government 2014; Council for the Curriculum, Examinations and Assessments 2019; Welsh Government 2021). However, little is known how to deliver RSE effectively to students with intellectual disability, and, importantly, how to measure the impact of RSE in schools. Families are very concerned about children's safety, and teachers are unclear on what RSE should achieve in the school environment, and how to get parents on board with its aims and objectives (Todd 2009; Lafferty *et al*. 2012; Pownall *et al*. 2012).

Existing systematic reviews on RSE have focused on its content, delivery, and effectiveness for people with intellectual disability of any age and have only included academic papers written in English (Schaafsma *et al*. 2015; McCann *et al*. 2019; Sala *et al*. 2019; Brown *et al*. 2020). These reviews have suggested that existing RSE programmes lack specific outcome goals and that outcomes measured are heterogeneous (e.g. some studies measured knowledge of sexuality topics and some self‐protection skills). This inconsistent use of RSE outcomes in the literature hinders the comparison of the effectiveness of RSE curricula across studies which in turn could affect the development of appropriate RSE for this vulnerable population. In addition, researchers highlight that people with intellectual disability are rarely involved in the development of RSE and thus their needs and concerns are not considered (Schaafsma *et al*. 2015; McCann *et al*. 2019; Sala *et al*. 2019). Instead, researchers are the ones to decide what outcomes to select in their evaluation studies to assess the effectiveness of RSE for students with intellectual disability.

Given these important limitations, there is an urgent need to clearly map the outcomes of RSE for students with intellectual disability whilst also achieving engagement by all stakeholders (students with intellectual disability, parents, teachers, policy makers, and researchers) in this process. The development of a Core Outcome Set (COS) involves identifying ‘what’ to measure and includes stakeholders' opinions on what constitutes meaningful outcomes (Williamson *et al*. 2012). The COS provides a minimum standard of outcomes that all trials, evaluation studies and practice‐based audits should measure and report within a specific health or social care area (Williamson *et al*. 2012). The Core Outcome Measures in Effectiveness Trials (COMET) Initiative proposes a standardised process, where the first step of COS development, the systematic review, aims to identify outcomes measured in quantitative studies and to identify stakeholders' perspectives on outcomes reported in qualitative studies (Williamson *et al*. 2017). These outcomes will then form an inclusive ‘long list’ of potential outcomes for later parts of the process (Williamson *et al*. 2017). An important aspect of such a systematic review involves identifying all outcome measurement instruments used in the literature and evaluating their psychometric properties (Prinsen *et al*. 2016). The COnsensus‐based Standards for the selection of health Measurement Instruments (COSMIN) Initiative has developed criteria for evaluating instruments' measurement properties (reliability, validity, and responsiveness) that help to select the most reliable instruments to measure outcomes (Mokkink *et al*. 2018).

To date, there is no published COS of RSE for students with intellectual disability. The development of such a COS will help to develop consensus in this sensitive area and would also provide, for the first time, a standardised set of outcomes to be used in research and educational practice to assess RSE delivery and develop effective education for this population. Therefore, we aim to develop a COS for RSE for students with intellectual disability for use in English educational and research settings. This paper reports findings of a systematic review that was carried out as a first step in this COS development. The objectives of the review were (1) to identify outcomes of RSE for students with intellectual disability reported in existing studies; (2) to identify measurement instruments used to measure RSE outcomes; (3) to evaluate the identified instruments' measurement properties (validity, reliability and responsiveness) using COSMIN criteria (Mokkink *et al*. 2018).

## Methods

The systematic review was registered with PROSPERO (registration number: 1787) and carried out following PRISMA guidelines (Liberati *et al*. 2009). The entire COS study is registered prospectively in the COMET database (registration number: CRD42021243176).

### Search strategy

The search consisted of two stages. The first stage of the search was carried out in March 2021 to identify all outcomes of RSE and their measurement instruments using the electronic databases and grey sources listed in Table 1. Citation and reference searching was also performed. The search included a combination of controlled and free‐text terms related to intellectual disability, the age of the population and RSE, which were combined using the Boolean operator ‘AND’ and adapted for each database (see Table S1 in the supporting information).

**Table 1:** Sources used

	Sources
Electronic databases	MEDLINE (Ovid provider), Embase (Ovid provider), PsychINFO (Ovid provider), Social Science Citation Index in the Web of Science database, ERIC (EBSCO provider), BEI (British Education Index; EBSCO provider), ASSIA (Applied Social Sciences Index and Abstracts; ProQuest provider), IBSS (International Bibliography of the Social Sciences; ProQuest provider) and SCOPUS.
Grey sources	Research gate, Google Scholar (first 10 pages), Google (first 10 pages), NICE Evidence Search, The Kings Fund, Zetoc, WorldCat, OpenGrey and EThoS.
Other sources	Cochrane Library and Clinicaltrials.gov websites.

The second stage of the search was carried out in August 2021 to retrieve studies on identified instruments' measurement properties using the same databases and grey sources as in the first stage. For each instrument the search was carried out separately and included the name of the instrument and terms on intellectual disability which were combined with the Boolean operator ‘AND’ and adapted for each database (see Table S1 in the supporting information). We also planned to include in the search the COSMIN filter (Terwee *et al*. 2009) developed to support retrieval of studies on instruments' measurement properties but in the end it was not included in the final search as no studies were retrieved using this filter.

### Inclusion and exclusion criteria

In the first stage of the review, articles were eligible for inclusion if they met the following criteria:

1. at least 75% of participants were students with intellectual disability aged 5–25 years or parents, teachers, and school staff of students with intellectual disability aged 5–25 years. Studies were included if intellectual disability was administratively defined as well as studies that defined intellectual disability using standardised assessments of IQ and/or adaptive functioning. Students with co‐occurring conditions (e.g. autism) in addition to intellectual disability were eligible for inclusion. The upper age limit of 25 years was selected to include young adults still in education (e.g. special schools that serve students until the age of 25);
2. the study was about any type of RSE that is delivered in schools, home, and social care settings. Studies that focused on clinical interventions to treat clinical problems related to sexuality that are not part of an educational curriculum were excluded;
3. contained information on outcomes measured after RSE delivery or stakeholders' views of RSE content;
4. used any qualitative, quantitative, observational, and mixed‐methods study designs. Secondary research, for example, systematic reviews were excluded.
5. published from 1999 in any language. Searches were from 1999 onwards because most of the development of RSE policies in Europe took place at this time (Ketting and Ivanova 2018) as well as their integration in education (Department for Education and Employment 2000).

In the second stage of the review, articles were eligible for inclusion if (1) at least 75% of the sample consisted of students with intellectual disability aged 5–25 years and (2) the study provided information on the instrument's development or measurement properties (e.g. reliability and validity) or evaluated the interpretability of scores (e.g. distribution of scores or floor and ceiling effects).

### Study selection and data extraction

In both stages of the review, retrieved studies were stored in EPPI‐Reviewer 4 (Thomas *et al*. 2020) and checked for duplicates. Screening of titles/abstracts and full‐texts was performed by the main reviewer (LP). Twenty per cent of titles and full‐text records were independently screened by a second reviewer (AP). We recorded numbers and reasons for excluding studies. Any disagreements or discrepancies were resolved through discussion with the senior review team (CV and VT). Studies written in non‐English languages that had an abstract written in English were screened against the inclusion criteria; we did not exclude any studies based on whether they had an English abstract or not. The full text of articles written in a non‐English language where the abstract appeared to meet the criteria or where there was no abstract were screened by the review team who were proficient or native‐level fluent in the relevant language. Other researchers and PhD students (mentioned in the acknowledgments section) helped to screen studies written in a language the review team was not fluent in. We also used professional translation services for some studies that the core review team was unable to translate.

Data were extracted by the main reviewer (LP) and 10% of the data were also independently extracted by the second reviewer (AP) to check reliability. In the first stage of the review, data were extracted on study characteristics, sample, data collection method, RSE, outcomes and outcome measurement instruments. In the second stage of the review, we planned to extract data on: 1) study characteristics; 2) results on the instruments' measurement properties (validity, reliability, and responsiveness) and 3) information on the interpretability of scores (such as the distribution of scores or floor and ceiling effects) and instruments' feasibility characteristics (e.g. cost of the instrument, required equipment or training). However, this was not possible as no studies retrieved met the inclusion criteria.

### Risk of Bias

In the first stage of the review, a risk of bias assessment was not planned as it was not relevant to the research question. In the second stage of the review, we planned to use the COSMIN Risk of Bias checklist (Mokkink 2017) to evaluate risk of bias, but this was not performed as no study retrieved met the inclusion criteria.

### Outcome categorisation

We used the COMET Handbook (Williamson *et al*. 2017) for categorising the extracted outcomes into a long list. An ‘outcome’ was defined as: any construct measured following RSE delivery, stakeholders' views of RSE content, any RSE‐related construct that was part of the education delivered to students with intellectual disability.

First, outcomes were extracted verbatim from the papers with their definitions (if available) and compiled into a list. The main reviewer (LP) grouped the verbatim outcomes by educational stage into three separate lists. RSE outcomes reported for/by students aged 5–10 years were grouped into a list of outcomes for primary education, outcomes reported for/by students aged 11–16 years were grouped into a list of outcomes for secondary education and outcomes reported for/by students aged 16–25 years were grouped in a list of outcomes for further education. Then, the outcomes in those three lists that were overlapping or had the same definitions were merged under the same outcome name by the main reviewer (LP), recording the frequency this outcome was reported in the studies and who reported this outcome (e.g. students with intellectual disability, parents, teachers and/or whether it was measured by researchers). The outcomes that were considered semantically related by the main reviewer (LP) were presented to the senior review team (CV and VT) for evaluation and after that were grouped into outcome domains. The senior review team (CV and VT) also reviewed the lists and outcome categorisation. The feasibility and measurability of identified outcomes was also reviewed independently by each core reviewer (LP, CV, and VT) (see Table 2 for the criteria). Any disagreements or discrepancies about outcomes were resolved by consensus through discussion with another reviewer.

**Table 2:** Criteria used for assessing feasibility and measurability of identified outcomes

Outcomes rated by two reviewers independently as one of the following options were not included in the lists:
(1) This outcome does not have a definition, we cannot provide one, and it is unclear what this outcome is referring to.
(2) This outcome is not a directly relevant outcome of RSE (meaning that the identified outcome is not related to the RSE topic, for example, a clinical outcome such as an incidence of hyperactivity during the RSE teaching).
(3) This outcome is not student centred (meaning the identified outcome does not refer to aspects associated with students attitudes, knowledge or skills development but relates to other aspects, for example, school environment or teachers characteristics).
(4) This outcome is not a feasible outcome to be used in the educational setting (meaning that there are contextually insurmountable logistic barriers to implementation, for example, follow‐up period does not allow to be measured in educational settings).

### Evaluation of measurement properties of identified instruments

In the second stage of the review, we planned to apply the COSMIN criteria for content validity (Terwee *et al*. 2018) to appraise studies on the development of an instrument and content validity evaluations by two reviewers independently. The COSMIN criteria for good measurement properties (Mokkink *et al*. 2018) would have been applied to rate the other measurement properties of identified studies by two reviewers independently as well. However, this was not performed because none of the retrieved studies met the inclusion criteria as discussed in detail in the results section.

## Results

### First stage of the review

**Figure 1:**
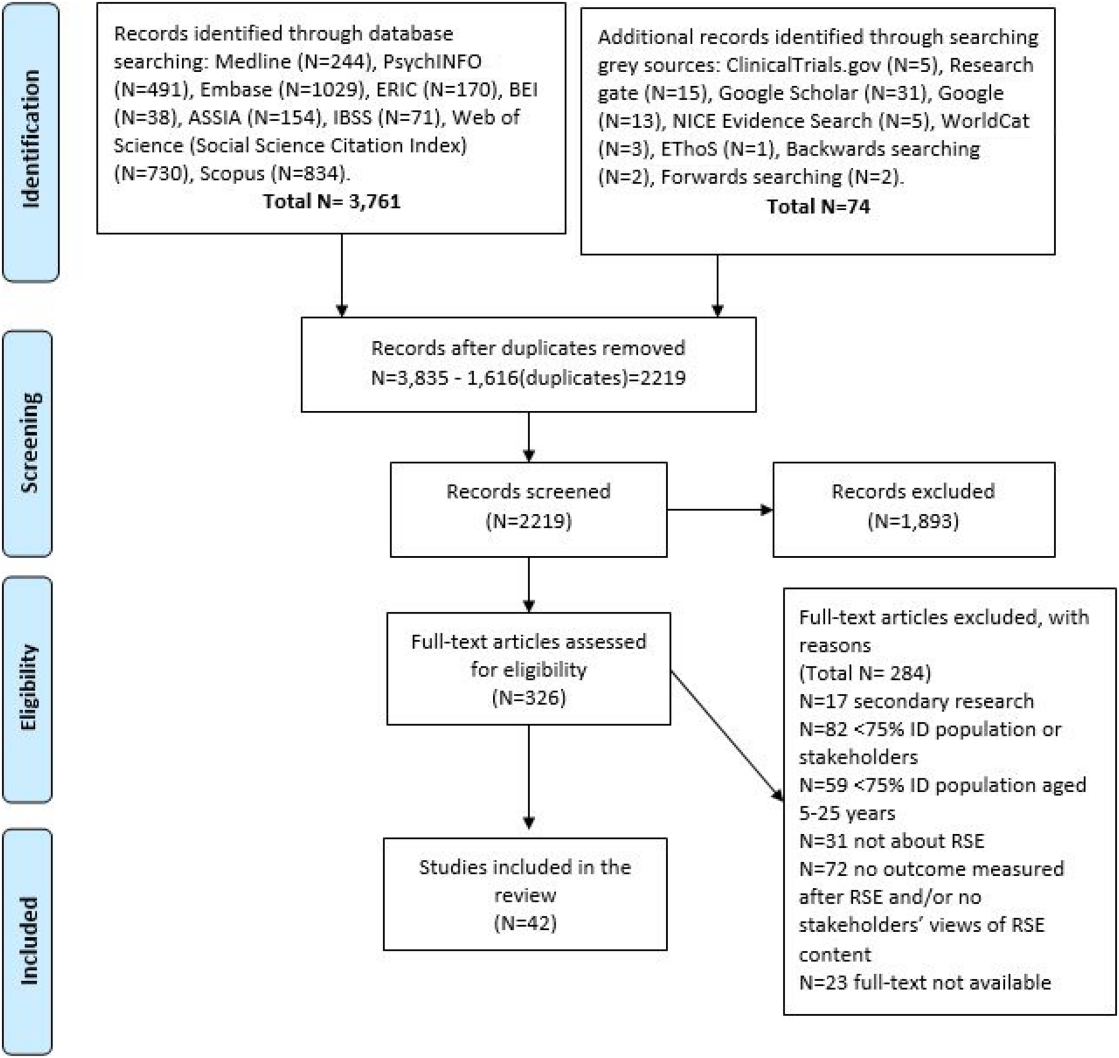
PRISMA flow diagram. [Colour figure can be viewed at wileyonlinelibrary.com]

The search retrieved 2219 unique articles of which 326 articles were screened on full text. A total of 284 articles were excluded as not meeting the inclusion criteria. Out of those papers that did not meet the criteria, 17 were not written in English, for example, French, Arabic, Turkish, Polish, Portuguese and Spanish. A total of 42 articles were included in the review. Please see Figure 1 for the PRISMA flow diagram.

### Study characteristics

Table 3 provides a description of the 42 included studies. Out of these, 27 studies provided qualitative data, six quantitative data and nine mixed‐methods data. Studies were conducted in the UK, USA, Africa, Turkey, Australia, Sweden, Ireland, Spain, Greece, Cyprus, Lithuania, Iran, India, Israel, Japan, and Republic of North Macedonia. Papers were written in English, Lithuanian, and Spanish.

**Table 3:** Characteristics of the included studies grouped by type of data provided

Study reference	Study location, language of report	Aim	Data collection method	Sample	Setting
*Qualitative data*					
Aderemi (2014)	Location: Nigeria (South Africa) Language: English	Explore educators opinions of sexuality and sexuality education for learners with ID.	Semi‐structured interviews	12 teachers (3 male and 9 female) aged 29–55 years of students with ID. ID: age, gender, level of ID not reported.	Special schools
Finlay *et al*. (2015)	Location: UK Language: English	Examine how Sexual Health Education is delivered to people with ID.	Semi‐structured interviews and observations of SHE sessions	4 teachers (age and gender not reported) of students with ID. ID: students aged 16–19 years with mild–moderate ID, gender not reported.	Youth club and special school
Frawley and Wilson (2016)	Location: Australia Language: English	Explore views of people with ID of sex education.	Focus groups	25 individuals with ID (14 male and 11 female), aged 17–20 years (Mean age = 18.6 years). ID: level of ID not reported.	Community transition program
Gokgoz *et al*. (2021)	Location: Turkey Language: English	Explore experiences of mothers providing sexual education to their children with Down syndrome.	Semi‐structured interviews	7 mothers of children with Down syndrome. ID: 3 male and 5 female children with Down syndrome aged 16–30 years (Mean age = 23.7 years) with mild to moderate ID.	Special education and rehabilitation centre
Garwood and McCabe (2000)	Location: Australia Language: English	Evaluate the effectiveness of 2 different sex education programs delivered to people with ID.	Questionnaires	6 males with ID: 3 in Co‐Care program aged 12–25 years (Mean age = 19 years, SD = 6.5) and 3 in Family Planning Victoria program aged 28–32 (Mean age = 31.6 years, SD = 3.2). ID: mild ID.	Community service

Study reference	Study location, language of report	Aim	Data collection method	Sample	Setting
Girgin‐Büyükbayraktar *et al*. (2017)	Location: Turkey Language: English	Explore teachers opinions of providing sex education for mentally handicapped students.	Semi‐structured interviews	25 teachers (18 female and 7 male) aged 25–40 years of students with ID. ID: age and level of ID not reported.	Special education and rehabilitation centres
Goli *et al*. (2020)	Location: Iran Language: English	Explore experiences of girls with ID, parents, teachers, and service providers of the sexual and reproductive health of girls with ID.	Semi‐structured interviews and a focus group	52 participants: 4 girls with ID, 23 parents, 7 teachers, 16 healthcare providers and 2 school managers. ID: Girls aged 14–18 years, level of ID not reported.	Special schools
Hanass‐Hancock *et al*. (2018)	Location: South Africa Language: English	Explore experiences of teachers of implementing sex education for learners with ID.	Interviews (type of interviews not reported)	8 teachers (age and gender not reported) of students with ID. ID: age, gender and level of ID not reported.	Special schools
Karellou (2017)	Location: Greece Language: English	Explore attitudes of individuals with ID towards sexuality.	Structured interviews	66 people with ID (48 male and 18 female), 78.8% aged 15–25 years and 21.2% aged 26–30 years. ID: 36 (54.5%) moderate ID, 19 (28.8%) mild and 11 (16.6%) borderline ID.	Vocational training facility
Kostigen (2020)	Location: USA Language: English	Explore policies and teaching practices of sexuality education for students with ASD and ID.	Participant observations, semi‐structured interviews, and document reviews of policies at school	11 special school staff (2 male and 9 female): 7 behaviour therapists, 3 administrators and 1 clinician (works in special school). Participants age not reported. ID: school staff serve 45 students with ASD and ID, 7 girls (15%) and 38 (85%) boys, aged 7–21 years (75% aged 14–21 years). ID level is reported as ‘low cognitive functioning’.	Special school

Study reference	Study location, language of report	Aim	Data collection method	Sample	Setting
Kürtüncü and Kurt (2020)	Location: Turkey Language: English	Examine the opinions of mothers of sexual education of children with ID.	Semi‐structured interviews and a questionnaire	14 mothers (Mean age = 39.2 years) of children with ID. ID: children (71.4% males and 28.6% females) aged 10–15 years (Mean age = 12.3 years). 42.9% had Down Syndrome, 57.1% had ID, ASD, speech, hearing and physical disabilities.	Education and rehabilitation centre
Lafferty *et al*. (2012)	Location: Northern Ireland (UK) Language: English	Examine the attitudes of family carers, direct care staff and trained professionals of the provision of RSE for teenagers and adults with ID.	Focus groups and semi‐structured interviews	Focus groups: 26 carers (fathers, sisters, brothers, and sisters‐in‐law) and 24 staff members (23 female and 1 male) from day centres and supported accommodation services. Interviews: 22 carers and 24 staff members (15 female and 9 male). ID: teenagers and young adults with ID, gender and level of ID not reported.	Community service
Löfgren‐Mårtenson (2012)	Location: Sweden Language: English	Explore experiences of adolescents with ID receiving of sex education.	Interviews (type of interviews not reported)	16 students with ID (7 male and 9 female) aged 16–21 years. ID: level of ID reported as ‘varying degrees of ID’.	Special schools
Menon and Sivakami (2019)	Location: India Language: English	Explore parents perceptions and concerns about the sexuality and reproductive health of their child with IDD.	Semi‐structured interviews	14 primary caregivers (9 mothers and 1 guardian) of individuals with IDD. ID: 4 children had ASD, 2 had ASD and ID, 5 had ID and mutism and 2 had Down Syndrome. 1 child was aged <10 years, 7 aged 10–20 years and 5 aged 21–30 years.	Special schools

Study reference	Study location, language of report	Aim	Data collection method	Sample	Setting
Nelson *et al*. (2020)	Location: Sweden Language: English	Explore teachers experiences of teaching Sexual and Reproductive Health and Rights Education to students with ID.	Semi‐structured interviews	10 teachers (6 female and 4 male) aged 24–55 years (Mean age = 39 years) of students with ID. ID: students aged 16–20 years with mild–severe ID.	Special schools
Peters (2007)	Location: USA Language: English	Explore attitudes of teachers towards the teaching of sexuality education to students with mental retardation.	Survey	90 special education teachers (73 female and 7 male) of students with mental retardation. ID: Teachers taught students aged 5–19 years. Students had mild–severe ID and other disabilities: autism, speech and language impairments, visual impairments and specific learning disabilities.	Special school
Phasha and Runo (2017)	Location: Kenya (Africa) Language: English	Explore sexuality education offered to learners with ID.	Semi‐structured interviews and focus groups	56 students with ID (32 male and 24 female) aged 16–22 years and 15 school staff: 5 head teachers and 10 teachers (gender not reported). ID: mild ID.	Special school
Pownall *et al*. (2011)	Location: Scotland (UK) Language: English	Explore mothers experiences of dealing with the sexuality of offspring with and without ID.	Semi‐structured interviews	8 mothers aged 46–53 years (Mean age = 48 years, SD = 2.4) of children with ID and without ID. ID: 4 males and 4 females with ID aged 17–19 years (Mean age = 18 years, SD = 0.8); 1 had Cerebral palsy, 2 Down syndrome; 1 Visual‐perceptual and spatial disability; 3 Global developmental delay and 1 ASD.	Participants home
Pryde and Jahoda (2018)	Location: Scotland (UK) Language: English	Explore views of mothers on sexuality and sexual development of their sons with ASD and ID.	Semi‐structured interviews	5 mothers of children with ID and ASD. ID: 5 males with moderate–severe ID and ASD aged 16–24 years.	Not specified

Study reference	Study location, language of report	Aim	Data collection method	Sample	Setting
Todd (2009)	Location: UK Language: English	Observe the delivery of sex education in a special school.	Observations of a classroom (type of observation is not reported) and interviews (type of interviews not reported)	Observations: a class of children with ID aged 15–16 years in a special school for students with severe ID, physical disabilities, and sensory impairments. Level of ID varied in the class and not all had severe ID. Interviews‐ teachers (no other information provided).	Special school
Ustilaitė and Petrauskienė (2018)	Location: Lithuania Language: Lithuanian	Explore parents experiences and needs when providing sex education to their children with ID.	Focus group	8 mothers aged 36–58 years of children with ID. ID: moderate level of ID, aged 12–21 years (5 male and 3 female).	Community service
Wilkenfeld and Ballan (2011)	Location: USA Language: English	Explore educators attitudes and beliefs of the sexuality of individuals with developmental disabilities.	Semi‐structured interviews	5 teachers (1 male and 4 female) aged 42–57 years (Mean age = 50.2 years) from special schools, 5 instructors (1 male and 4 females) aged 30–50 years, Mean age = 37 years) from adult day services. ID: teachers of middle‐high school aged students with ID, with ID level ranging from <12 months (i.e. sensory level of function) to IQs < 70. All students had multiple physical disabilities in addition to ID, for example, Cerebral Palsy and ‘various genetic disorders’ (which are not specified).	Special schools and adult day services

Study reference	Study location, language of report	Aim	Data collection method	Sample	Setting
Williams *et al*. (2014)	Location: Scotland (UK) Language: English	Identify experiences of people with ID about sexual health services.	Structured interviews	34 people with ID (17 male and 17 female) aged 16–35 years (64% aged 16–17 years). ID: level of ID not reported.	Special schools, colleges, day, and residential services
Williamson (2017)	Location: USA Language: English	Explore experiences of women with ID about sexuality and sex education.	Semi‐structured interviews and focus groups	6 women with ID and ASD aged 19–21 years. ID: mild to moderate ID (IQ = 54–70).	Special school
Wiseman and Ferrie (2020)	Location: Scotland (UK) Language: English	Explore experiences of women with ID of receiving Relationship Sexual Health and Parenthood Education.	Focus groups and a questionnaire	21 women with ID completed the questionnaire and 12 women with ID in focus groups, aged 18–25 years (the sample involved older women; however, results for this age group were extracted). ID: level of ID not reported.	Questionnaire‐online; focus groups‐ not specified.
Wright (2011)	Location: UK Language: English	Explore whether young people with ID receive sex education.	Semi‐structured interviews and focus groups	7 people with ID aged 11–16 years (gender not reported) without physical impairments or ASD, 7 parents, 12 teachers and 2 nurses from a special school. ID: level of ID not reported.	Special school
Yektaoglu‐Tomgüsehan and Akçamete (2018)	Location: Cyprus Language: English	Examine the opinions of teachers of sex education for students with mental deficiencies.	Semi‐structured interviews	6 teachers (5 female and 1 male) aged 26–30 years of students with ID. ID: level of ID and students age not reported.	Special school
*Quantitative data*					

Study reference	Study location, language of report	Aim	Data collection method	Sample	Setting
Dukes and McGuire (2009)	Location: Ireland Language: English	Evaluate a sex education intervention for people with ID.	Design: Single subject multiple baseline Method: Questionnaires	4 adults with ID (2 male and 2 female) aged between 22 and 23 years. ID: moderate level of ID (IQ = 41–45; Adaptive behaviour composite = 37–54). Diagnostic tools used: Vineland Adaptive Behaviour Scales and the Stanford‐Binet Intelligence Scale.	Community group homes
Holmes and Himle (2014)	Location: USA Language: English	Understand what topics were the most frequently discussed by parents when providing sex education to children with ASD based on the level of the childs functioning.	Design: Cross‐sectional Method: Survey	36 parents of children with ID and ASD (that meet the inclusion criteria in terms of child having ID). ID: children aged 12–18 years (Mean age = 15.5 years, SD = 1.9) with mild‐profound ID and ASD.	Online
Howard‐Barr *et al*. (2005)	Location: USA Language: English	Explore teachers opinions of what are the most and least important RSE topics to provide to students with ID.	Design: Cross‐sectional Method: Questionnaires	26 teachers of students with ID. 49% of teachers taught elementary school, 32% middle school and 19% high school. ID: level of ID not reported.	Special school
Luque and Lugo (2015)	Location: Spain Language: Spanish	Evaluate Emotional‐Sexual Education for students with ID.	Pre‐post evaluation Method: Questionnaires	8 students with ID (5 male and 3 female) aged 16–22 years from a special school (that meet the inclusion criteria). ID: level is not reported.	Special Education Centre
Plaks *et al*. (2010)	Location: Israel Language: English	Evaluate Social Sexual Group Education Program delivered to adolescents with ID and their parents.	Design: Single group pre‐post evaluation Method: Questionnaires	10 adolescents with neurogenetic conditions (5 male and 5 female) aged 15–24 years (Mean age = 19.5 years, SD = 2.6) and 14 parents (age, gender not reported). ID: level of ID not reported; 5 had Williams Syndrome, 3 Velocardiofacial syndrome, 1 neurofibromatosis, and 1 idiopathic ID.	Behavioural Neurogenetics Centre
Tsutsumi (2009)	Location: Japan Language: English	Explore teachers and parents opinions about sex education for mentally retarded pupils.	Design: Cross‐sectional Method: Survey	235 teachers (149 female and 86 male) and 312 parents (166 female and 43 male) of mentally retarded pupils. ID: level of ID not reported, students were aged 7–16 years.	Special classes in mainstream schools

Study reference	Study location, language of report	Aim	Data collection method	Sample	Setting
*Mixed methods data*					
Bleazard (2010)	Location: Western Cape (South Africa) Language: English	Explore attitudes of women with ID, teachers, and mothers towards the sexuality of women with ID.	Semi‐structured interviews, focus group and questionnaire (quantitative questions)	21 women with ID aged 16–25 years with visual, motor, speech, and growth difficulties in the interviews; 10 out of 21 women with ID also took part in a focus group, 10 parents in separate interviews and 12 teachers completed a questionnaire. ID: level of ID is not reported.	Special schools
Box and Shawe (2014)	Location: UK Language: English	Evaluate a sexuality and relationship programme delivered to adults with ID and explore their experiences of attending the attending the programme..	Semi‐structured interviews, participant observations, and pre‐post evaluation using a questionnaire.	2 male adults with ID (that meet the inclusion criteria) aged 20–24 years. ID: mild to moderate ID. 1 participant had mild ID, Aspergers syndrome, and ADHD. The other participant had moderate ID and ASD.	Community ID Service
Graff *et al*. (2018)	Location: USA Language: English	Evaluate sex education curriculum delivered to students with ID.	Questionnaires and open‐ended questions	53 students with IDD aged 18–27 years (Mean age = 20.7 years). 1^st^ intervention group: 13 students (46.2% males and 53.8% female), Mean age = 20.7 years, SD = 1.4, 76.9% had ID and 23.1% had ASD. 2^nd^ intervention group: 12 students (66% males and 33% females), Mean age = 20.8 years, SD = 1.7, 50% had ID and 50% ASD. 4th intervention group: 16 participants (43% males and 56% females), Mean age = 19.3 years, SD 4.6, 71% had ID and 29% ASD. The control group: 12 students with IDD (33.3% males and 66.6% females), Mean age = 22.1 years, SD = 21.9. 83.3% had ID and 16.7% other disabilities. ID: level of ID not reported.	Post‐secondary program for students with IDD at a university

Study reference	Study location, language of report	Aim	Data collection method	Sample	Setting
Kammes (2019)	Location: USA Language: English	Examine how parental attitudes and experiences towards sexuality differ for their children with ID and without ID.	Semi‐structured interviews and survey (quant questions)	50 parents (46 mothers, 4 fathers) of children with and without ID in the survey and 20 of parents (1 father and 17 mothers) in the interview. ID: 50 adult children with ID (30 male and 20 female) aged 18–33 years, Mean age = 23.9 years, SD = 3.7 with other co‐morbidities, for example, Down syndrome, ASD, WS, physical, mental health and speech problems.	Online survey and phone or video interview
Pownall *et al*. (2012)	Location: Scotland (UK) Language: English	Explore mothers experiences of providing sex education to children with ID and without ID.	Questionnaire with quant and qual questions	30 mothers (Mean age = 48.8 years, SD = 6.5) of young people (15 males and 15 female) with ID (Mean age = 18.8 years, SD = 1.9). ID: children were aged 16–24 years (Mean age = 18.8 years, SD = 1.9) and had mild ID. 4 children had physical disability (not specified) in addition to ID.	Participants homes
Rohleder *et al*. (2012)	Location: Western Cape (South Africa) Language: English	Explore the extent to which safe sex education is being provided to young people with disabilities.	Survey (quant questions), semi‐structured interviews, and a focus group	55 teachers of students with various disabilities ID: 76.5% of teachers served students with ID, level of ID not reported.	Special schools

Study reference	Study location, language of report	Aim	Data collection method	Sample	Setting
*Scottish Commission for Learning Disability* (2018)	Location: Scotland (UK) Language: English	Gather information about RSHPE provision in secondary schools for students with ID.	Survey (quant and qual questions)	130 school staff members that serve students with ID: 71.8% worked in mainstream school with additional support need unit, 25.2% worked in additional support need school, 1.9% worked in residential schools. ID: ID level is not reported.	Secondary mainstream schools and special schools for students with ID
Sheppard (2006)	Location: Australia Language: English	Evaluate the effectiveness of ‘Growing Pains’ program for students with ID and to explore parents and teachers views about the program.	Pre‐post evaluation using a checklist and questionnaires with quant and qual questions	68 students with ID (48 male and 20 female), 12 teachers and 13 parents. ID: mild to moderate ID, aged 11–15 years.	Special school
Stankova and Trajkovski (2021)	Location: Republic of North Macedonia Language: English	Assess the effects of the use of Social Stories to implement sexuality education with participants with ASD and cognitive difficulties.	Pre‐post evaluation and semi‐structured interviews	1 boy with ID, ASD and speech delay (that meets the inclusion criteria and results reported separately) aged 11 years. ID: level is reported as ‘low’.	Special school

ID, intellectual disability, ASD, autism spectrum disorder, IDD, intellectual and developmental disability, RSHPE, relationships, sexual health and parenting education, SHE, sexual health education.

A total of 1767 participants participated in the included studies: 38% (678) were teachers and school staff, 34% (612) were parents and caregivers, 23% (413) were students with intellectual disability and 3% (64) were staff members from community intellectual disability services. Out of 413 students with intellectual disability included, 211 were male and 202 were female, aged 5–25 years (73% were aged 16–25 years), 51% had mild to moderate intellectual disability. Two studies focused on students with genetic conditions (e.g. Down syndrome, Williams Syndrome, Velocardiofacal syndrome and neurofibromatosis) (Plaks *et al*. 2010; Gokgoz *et al*. 2021) and 4 studies involved students with intellectual disability with co‐occurring conditions (e.g. autism, ADHD, speech delay and physical disabilities) (Bleazard 2010; Box & Shawe 2014; Williamson 2017; Stankova & Trajkovski 2021).

### RSE characteristics

Out of 42 included studies, 12 studies reported some RSE intervention characteristics (e.g. information on the population it was developed for, content, delivery, setting and/or providers) delivered to students with intellectual disability (Garwood & McCabe 2000; Sheppard 2006; Dukes & McGuire 2009; Todd, 2009; Plaks *et al*. 2010; Box & Shawe 2014; Finlay *et al*. 2015; Luque & Lugo 2015; Williamson 2017; Graff *et al*. 2018; Kostigen 2020; Stankova & Trajkovski 2021) (see Table 4). The aim of the RSE programme was only reported in four studies (Sheppard 2006; Todd, 2009; Plaks *et al*. 2010; Graff *et al*. 2018). Only four studies specified that RSE was based on resources developed specifically for people with intellectual disability (Sheppard 2006; Dukes & McGuire 2009; Box & Shawe 2014; Williamson 2017). In the retrieved studies RSE coverage included: the human body (private parts for males and females), puberty, hygiene, sexual activities and their consequences, contraception, different types of relationships, appropriate and inappropriate social and sexual behaviours, information on abuse and keeping safe. Across the studies, the content mainly focused on protection and managing risks rather than being more comprehensive with a balance of risk management and other skill development.

**Table 4:** RSE characteristics reported in the studies

Reference	RSE characteristics
Box and Shawe (2014)	**Name**: Sexuality and Relationships group based on programmes developed for people with ID (McCarthy & Thompson 1998; Craft 2003; Kerr‐Edwards & Scott 2003) **Aim**: Not reported. **Population**: Adult males with mild–moderate ID and ASD and ADHD aged 20–24 years. **Content**: Basic anatomy and body differences, puberty, hygiene, menstruation, menopause, sexual activities including same‐sex relationships, conception, contraception, safe sex including abstinence, masturbation, wet dreams, self‐examination, attractions, different types of relationships, forming and managing relationships, emotions, attitudes including stereotyping, good and bad touch, consent, public and private places, abuse, and assertiveness. **Delivery**: 10 group sessions of 2 h session once per week. **Setting**: Day centre for adults with ID. **Parent involvement**: Not reported. **Providers**: 1 male and 1 female community ID nurse.
Dukes and McGuire (2009)	**Name**: Sex Education based on the ‘Living Your Life – The Sex Education and Personal Development Resource for Special Education Needs’ (Bustard 2003) book. **Aim**: Not reported. **Population**: Adults (male and female) with moderate ID aged 22–23 years. **Content**: Sexual safety practices, physical self, sexual functioning, choices, and consequences in sexual matters. **Delivery**: Individual sessions of 45 min per session delivered twice per week. **Setting**: Group homes for adults with ID **Parent involvement**: Not reported. **Providers**: Researchers.
Finlay *et al*. (2015)	**Name**: Sex and Health Education. **Aim**: Not reported. **Population**: Adolescents (males and females) with mild–moderate ID aged 16–19 years. **Content**: Sexual behaviour, contraception, norms, and relationships. **Delivery**: Weekly group sessions (4–8 people) of 1 h per session (total number of sessions not reported). **Setting**: Youth club and special schools **Parent involvement**: Not reported. **Providers**: 4 (male and female) youth service workers
Garwood and McCabe (2000)	**Name**: Co‐Care. **Aim**: Not reported. **Population**: Males with mild ID aged 12–25 years (Mean age = 19 years, SD = 6.5). **Content**: Feelings, body language, social skills, the human life cycle, puberty, body awareness ‘private’ and ‘public’ behaviour, sexual relationships, conception, pregnancy, childbirth, contraception (including safe sex, STDs), menstruation and protective behaviours. **Delivery**: Total number of sessions not reported. Sessions were delivered twice per week for 45 min per session. **Setting**: Community health service **Parent involvement**: Not reported. **Providers**: 2 (male and female) educators.
Graff *et al*. (2018)	**Name**: Relationships and Sexuality Education. **Aim**: Teach skills and increase judgement about healthy relationships, sexuality, and safe boundaries. **Population**: Adults (male and female) with IDD (some had ID, and some had ASD) aged 18–27 years (Mean age = 20.7 years, SD = 1.4). Level of ID not reported. **Content**: Relationships and self‐awareness, maturation, the life cycle, sexual health, being strong, staying safe and hygiene. **Delivery**: Group sessions (total number of sessions and length of the session not reported). **Setting**: Inclusive postsecondary program for students with IDD at a university. **Parent involvement**: The curriculum was developed with guidance from parents. **Providers**: Teachers (gender not reported).
Kostigen (2020)	**Name**: Health and Sexuality Education. **Aim**: Not provided. **Population**: Students with ID and ASD aged 7–21 years (75% 14–21 years). Level of ID not reported. **Content**: Concepts of privacy, body part identification, hygiene skills, respecting personal space, and requesting another to stop an aversive activity. **Delivery**: Group sessions (total number of sessions and length of the session not reported). **Setting**: Special school. **Parent involvement**: Not provided. **Providers**: Teachers.
Luque and Lugo (2015)	**Name**: Emotional‐Sexual Education based on Affective Sex Education materials (Bolaños *et al.* 1994; Colectivo Harimaguada 2007). Not specified if these materials were developed for people with ID. **Aim**: Not reported. **Population**: Students with ID (5 male and 3 female) aged 16–22 years (level of ID not reported). **Content**: the education contained 6 parts: 1) ‘body’ which involved private body parts, understanding the anatomy and physiology of the genitals, body changes during adolescence, body differences between males and females; 2) ‘social skills’ which involved active listening, assertiveness, empathy, respect for other people and their desires; 3) ‘emotional’ which included work on relational skills and abilities such as recognition and expression of emotions, self‐esteem; 4) ‘affective‐sexual’ which focused on learning about different ways people express affection and feelings, understanding of contraceptive methods and masturbation; 5) ‘private/public context’ which includes awareness of the difference between public and private spaces; 6) ‘abuse or sexual abuse’ which included information on appropriate and inappropriate physical contact, learning to say ‘no’ and what to do in case of abuse. **Delivery**: Group sessions of 1 h per session, two sessions per week delivered over 4 months. **Setting**: Special Education Centre. **Parent involvement**: Not reported. **Providers**: Teachers.
Plaks *et al*. (2010)	**Name**: Social Sexual Education **Aim**: Adolescents self‐identification, acceptance of the developmental disability, independence in social life, establishment of friendship and intimate relationship, sexual knowledge and sexual development, safety skills and assertiveness skills. **Population**: Adolescents (male and female) with genetic conditions (WS, VCFS, ID) aged 15–24 years (Mean age = 19.5 years, SD = 2.6). Level of ID not reported. **Content**: Anatomic parts of the sexual system, differences between males and females sexual organs, sexual development, changes occurring in the body during adolescence, appropriate sexual behaviour (e.g. masturbation is something one does in private). **Delivery**: 10 group sessions (length per session not reported). **Setting**: Behavioural Neurogenetics Centre. **Parent involvement**: Parents participated in a separate Social Sexual Education group. **Providers**: Not reported.
Sheppard (2006)	**Name**: Growing Pains Program (based on variety of resources). **Aim**: Skills and knowledge acquisition in these areas: socialisation, personal hygiene, protective behaviours, sexuality, assertiveness, and substance abuse. **Population**: Adolescents with mild–moderate ID, aged 11–15 years. **Content**: Social skills: self‐esteem, anger, conflict management, peer interaction, rights and responsibilities, decision‐making; drug education; relationships and sexuality; protective behaviours: safe behaviours, public/private, body parts; grieving and loss; human life cycle; personal hygiene. **Delivery**: Group sessions delivered over 20 weeks (45–65 min per session). **Setting**: Special school **Parent involvement**: Not reported. **Providers**: Teachers (gender not reported).
Stankova and Trajkovski (2021)	**Name**: Sex Education curriculum using Social Stories. **Aim**: Not reported. **Population**: An adolescent male with ASD and low level of intellectual functioning aged 11 years with speech and developmental delay. ID level not reported. **Content**: 14 Social Stories: My body; Private and Public Space; My intimate parts of the body; Why do I wear clothes; Where can I be naked; How do I grow and change; Wet dreams; Masturbation; What is pleasant; What is unpleasant; Pleasant touches; Unpleasant touches; Types of relationships. Content was personalised based on individual needs and cognitive level. **Delivery**: Individual sessions of 45 min over 6 months (total number of sessions not reported). **Setting**: Special school. **Parent involvement**: Parents were asked about the perceived benefits in their children after they received the education. **Providers**: Teacher (gender not reported).
Todd (2009)	**Name**: Personal Relationships and Health Education. **Aim**: Develop respect for self and others, build up a positive self‐concept, encourage the concept of loving relationships within the context of the family and encourage socially acceptable behaviour. **Population**: Class of adolescents aged 15–16 years in a special school for children with severe ID. Level of ID varied in the class. **Content**: ‘Range of topics, from health to appropriate greeting patterns, and a brief biological tour of the body and sex education’. **Delivery**: Group sessions of 90 min delivered over 13 weeks. **Setting**: Special School. **Parent involvement**: Not reported. **Providers**: Teachers.
Williamson (2017)	**Name**: Sex Education curriculum based on ‘positive choices programme for people with ID’ (Duguay 2009) **Aim**: Not reported. **Population**: Women with ID and ASD aged 19–21 years with mild–moderate ID (IQ = 54–70). **Content**: Not reported. **Delivery**: Group sessions of 1.5 h per session delivered over 32 weeks (one per week). **Setting**: Special school. **Parent involvement**: Not reported. **Providers**: Teachers

Some studies reported delivering RSE in small groups whilst other studies delivered RSE on an individual basis for a period ranging between 10 weeks to 6 months. RSE was delivered in special schools, group homes, students' homes and community intellectual disability services (e.g. day centres, youth clubs and health services) by teachers, parents, youth service workers, community intellectual disability nurses and researchers. Only two studies reported that parents were involved in the development of RSE content delivered in schools (Sheppard 2006; Graff *et al*. 2018). None of the studies reported that parents were involved in the delivery of RSE in schools (e.g. asked to deliver after‐school activities to their children at home).

### Outcomes

A total of 135 individual outcomes were extracted from the studies. Outcomes were grouped by educational stage (e.g. secondary education and further education). Seven studies reported RSE outcomes for students with intellectual disability but did not specify their age (Howard‐Barr *et al*. 2005; Rohleder *et al*. 2012; Aderemi 2014; Girgin‐Büyükbayraktar *et al*. 2017; Hanass‐Hancock *et al*. 2018; *Scottish Commission for Learning Disability* 2018; Yektaoglu‐Tomgüsehan and Akçamete 2018). These outcomes were not included in the tables presented here but will still be included in the later stages of the COS process (see Table S2 in the supporting information for these outcomes).

### Outcomes for students in primary education

No studies reported outcomes specifically for students with intellectual disability in primary education (aged 5–10 years). However, three studies reported outcomes for students aged 5–25 years and the lower age of participants was in the primary education age range (see Table 5) (Peters 2007; Tsutsumi 2009; Menon & Sivakami 2019). Outcomes in these studies were: understanding of hygiene, the human body (e.g. what menstruation and wet dreams are), relationships (e.g. what dating, love and marriage is), human sexuality (heterosexuality, homosexuality and bisexuality), sex and its consequences (e.g. sexual intercourse and sexually transmitted diseases), appropriate social behaviour and ability to protect yourself from sexual abuse.

**Table 5:** RSE outcomes where the lower age of the sample involves students in primary education (5–10 years)

Outcome domain	Outcome	Age range	Reference	Who reported
Understanding of hygiene	Understanding of what personal hygiene is	7–16 years	Tsutsumi (2009)	Teachers and parents
Understanding of human body	Understanding of what menstruation is	5–19 years	Peters (2007)	Teachers
	Understanding of what wet dreams are	5–19 years	Peters (2007)	Teachers
Understanding of relationships	Understanding of what dating is	7–16 years	Tsutsumi (2009)	Teachers and parents
	Understanding of what marriage is	5–19 years	Tsutsumi (2009); Peters (2007)	Teachers and parents
	Understanding of love is	5–19 years	Peters (2007)	Teachers
	Understanding of what parenting is	5–19 years	Peters (2007)	Teachers
Understanding of human sexuality	Understanding of different sexualities (what heterosexuality, homosexuality, and bisexuality is)	5–19 years	Peters (2007)	Teachers
Understanding of sex and its consequences	Understanding of what pregnancy is	7–16 years	Tsutsumi (2009)	Teachers and parents
	Understanding of what contraception is	5–19 years	Tsutsumi (2009); Peters (2007)	Teachers and parents
	Understanding of what sexual intercourse is*	5–19 years	Tsutsumi (2009); Peters (2007)	Teachers and parents
	Understanding of what STDs are	7–16 years	Tsutsumi (2009)	Teachers and parents
Understanding of appropriate behaviour	Understanding of socially acceptable public behaviour	10–25 years	Menon and Sivakami (2019)	Parents
Understanding of sexual abuse	Ability to protect yourself from sexual abuse	5–19 years	Peters (2007)	Teachers

STDs, sexually transmitted diseases.

### Outcomes for students in secondary education

Table 6 provides 43 individual outcomes for students with intellectual disability in secondary education (aged 11–16 years) extracted from 15 studies and grouped under 11 outcome domains: understanding of the human life cycle, the human body, relationships, hygiene, keeping safe, appropriate and inappropriate behaviours, human sexuality, sex and its consequences, rights and responsibilities, emotional vocabulary, and positive self‐esteem.

**Table 6:** RSE outcomes reported for students in secondary education (11–16 years)

Outcome domain	Outcome	Frequency of reporting and study reference	Who reported/measured
Understanding of human life cycle	Able to identify the stages of the human life cycle (e.g. birth, baby, child, teenager, adult, elderly and death)	2 studies: Sheppard (2006); Stankova and Trajkovski (2021)	Measured by researchers
Understanding of human body	Understanding of private body parts for females and males	5 studies: Kürtüncü and Kurt (2020); Stankova and Trajkovski (2021); Sheppard (2006); Holmes and Himle (2014); Kostigen (2020)	Parents, school staff and measured by researchers
	Understanding of puberty related body changes for males and females	2 studies: Stankova and Trajkovski (2021); Karellou (2017)	Students with ID and measured by researchers
	Understanding of what wet dreams are	2 studies: Stankova and Trajkovski (2021); Peters (2007)	Teachers and measured by researchers
	Understanding of what erection is	1 study: Stankova and Trajkovski (2021)	Measured by researchers
	Understanding of what menstruation is^†^	1 study: Peters (2007)	Teachers
Understanding of hygiene	Understanding of what personal hygiene is	2 studies: Tsutsumi (2009); Holmes and Himle (2014)	Parents, teachers
	Hygiene skills (able to: dress self, undress self, wash hands and face, menstrual care, showering, toileting, grooming, e.g. hair and teeth)	2 studies: Kostigen (2020); Sheppard (2006);	School staff and measured by researchers
Understanding of relationships	Understanding of different type of relationships	3 studies: Wright (2011); Ustilaitė and Petrauskienė (2018); Stankova and Trajkovski (2021)	Students with ID and parents, measured by researchers
	Appropriate greeting patterns	1 study: Todd (2009)	Content of RSE
	Friendship skills	1 study: Sheppard (2006)	Measured by researchers
	Understanding of how to make close friends	1 study: Wright (2011)	Students with ID
	Understanding of how to socialise with girls/boys	1 study: Ustilaitė and Petrauskienė (2018)	Parents
	Understanding of what dating is	1 study: Tsutsumi (2009)	Teachers
	Understanding as to why people kiss	1 study: Ustilaitė and Petrauskienė (2018)	Parents
	Understanding of what love is	2 studies: Peters (2007); Ustilaitė and Petrauskienė (2018)	Teachers, parents
	Understanding of what marriage is	2 studies: Tsutsumi (2009); Peters (2007)	Teachers
	Understanding of what parenting is	1 study: Peters (2007)	Teachers
	Understanding of what sexual relationships are	1 study: Sheppard (2006)	Measured by researchers
	Ability to identify elements of peer pressure	1 study: Sheppard (2006)	Measured by researchers
	Ability to distinguish appropriate and inappropriate behaviour in different type of relationships	1 study: Stankova and Trajkovski (2021)	Measured by researchers
	Respecting yourself in romantic relationships (not sleeping with everyone)	1 study: Ustilaitė and Petrauskienė (2018)	Parents
Understanding how to keep safe	Understanding a concept of privacy	1 study: Kostigen (2020)	School staff
	Understanding of areas of a body that should not be touched by others	5 studies: Kürtüncü and Kurt (2020); Holmes and Himle (2014); Lafferty *et al*. (2012); Kostigen (2020); Stankova and Trajkovski (2021);	Parents, teachers, school staff and measured by researchers
	Understanding of what abuse is	1 study: Kürtüncü and Kurt (2020)	Parents
	Understanding of how to protect yourself from sexual abuse	2 studies: Peters (2007); Menon and Sivakami (2019)	Parents, teachers
	Protective behaviours (recognises safe/unsafe situations/environments, avoids of unsafe situations such as isolation, dark places, recognises threatening behaviour of others and knows what to do when someone shows threatening behaviour)	1 study: Sheppard (2006)	Measured by researchers
	Ability to request another to stop an aversive activity	1 study: Kostigen (2020)	School staff
Understanding of appropriate and inappropriate behaviour	Understanding public and private places	4 studies: Stankova and Trajkovski (2021); Holmes and Himle (2014); Lafferty *et al*. (2012); Sheppard (2006)	Parents and teachers and measured by researchers
	Ability to recognise public and private behaviour	1 stud: Sheppard (2006)	Measured by researchers
	Understanding as to why people wear clothes	1 study: Stankova and Trajkovski (2021)	Measured by researchers
	Understanding of appropriate sexual behaviour	1 study: Karellou (2017)	Students with ID
	Understanding of appropriate social behaviour	2 studies: Menon and Sivakami (2019); Wilkenfeld and Ballan (2011)	Parents, teachers
Understanding of human sexuality	Understanding of different sexualities (what heterosexuality, homosexuality, and bisexuality is)	1 study: Peters (2007)	Teachers
Understanding of sex and its consequences	Understanding of what masturbation is^‡^	3 studies: Stankova and Trajkovski (2021); Holmes and Himle (2014); Lafferty *et al*. (2012)	Parents, teachers
	Understanding of what pregnancy is	5 studies: Tsutsumi (2009); Lafferty *et al*. (2012); Ustilaitė and Petrauskienė (2018); Wilkenfeld and Ballan (2011)	Teachers, parents.
	Understanding of what contraception is	4 studies: Tsutsumi (2009); Karellou (2017); Lafferty *et al*. (2012); Peters (2007)	Teachers, parents, and students with ID
	Understanding of what sexual intercourse is^§^	4 studies: Tsutsumi (2009); Karellou (2017); Peters (2007); Ustilaitė and Petrauskienė (2018)	Teachers, parents, students with ID
	Understanding of what STDs are	3 studies: Tsutsumi (2009); Lafferty *et al*. (2012); Wilkenfeld and Ballan (2011)	Teachers, parents
	Understanding of consequences of sexual activities	2 studies: Ustilaitė and Petrauskienė (2018); Wilkenfeld and Ballan (2011)	Parents, teachers
Understanding of rights and responsibilities	Understanding of rights and responsibilities (demonstrates understanding of rights of self, demonstrates understanding of rights or others)	1 study: Sheppard (2006)	Measured by researchers
Emotional vocabulary	Understanding of different feelings and emotions (identifies feelings and emotions)	2 studies: Sheppard (2006); Lafferty *et al*. (2012)	Teachers and measured by researchers
Positive self‐esteem	Able to identify positive self‐attributes	1 study: Sheppard (2006)	Measured by researchers

STDs, sexually transmitted diseases; ID, intellectual disability.

^†^ One study contradict that this should be an outcome of RSE (Goli *et al*. 2020).

^‡^ Two studies contradict that this should be an outcome of RSE (Todd, 2009; Menon & Sivakami 2019).

^§^ One study contradicts that this should be an outcome of RSE (Menon & Sivakami 2019).

The most reported outcomes of RSE for students aged 11–16 years were: understanding of private body parts, understanding of areas of a body that should not be touched by others, and understanding what pregnancy is. Out of 43 extracted outcomes, 85% (36) of outcomes were knowledge based (e.g. understanding of what contraception is), 4% (2) of outcomes were skills based (e.g. friendship skills and hygiene skills), 9% (4) of outcomes were behaviour based (e.g. protective behaviour) and 2% (1) of outcomes were attitudes based (e.g. positive self‐esteem).

Out of the 15 studies, only two studies provided information about how some of the extracted outcomes were measured (Sheppard 2006; Stankova & Trajkovski 2021). In Stankova and Trajkovski (2021), understanding of sexuality was assessed before and after RSE delivery using pictures and asking students to say what pictures depicted or point to the correct pictures when asked. For example, students' understanding of areas of the body that should not be touched by others was assessed by presenting illustrations of people touching each other and asking students to say if this is appropriate touch or inappropriate touch based on people's facial expressions in the pictures. In Sheppard (2006), outcomes were measured based on teachers' reports of students' behaviour before and after RSE delivery. Teachers were asked to rate the frequency (always, regularly, occasionally, seldom, or never) students demonstrated friendship skills, understanding of what sexual relationships are, ability to identify elements of peer pressure, understanding of rights and responsibilities, understanding of different emotions and feelings, and ability to identify positive self‐attributes. However, what teachers were considering when evaluating these outcomes (e.g. what rights of self and rights of others actually involve and what specific skills were considered when evaluating friendship skills) and how these evaluations were conducted were not reported in the paper (e.g. whether teachers asked students to verbally describe positive self‐attributes or point to pictures).

### Outcomes reported by parents, teachers, and students in secondary education

There was an overlap between 13 (46%) out of 28 RSE outcomes reported by parents and teachers for students with intellectual disability in secondary education: understanding of personal hygiene, appropriate and inappropriate social and sexual behaviour, consequences of sexual activities, contraception, and protection from sexual abuse. Outcomes that were reported only by parents or teachers closely correspond. However, in three retrieved studies (Todd 2009; Menon & Sivakami 2019; Goli *et al*. 2020) parents and teachers did not agree that understanding of menstruation, masturbation, and sexual intercourse should be outcomes of RSE for students with intellectual disability.

Students with intellectual disability in secondary education (aged 11–16 years) reported six RSE outcomes: understanding of puberty‐related body changes for males and females, understanding of relationships (e.g. how to make close friends), and understanding of sexual intercourse (e.g. understanding of appropriate sexual behaviour and contraception).

### Outcomes for students in further education

A total of 92 individual outcomes for students in further education (aged 16–25 years) were extracted from 37 studies and grouped under 13 domains: understanding of the human body, hygiene, menstruation, human sexuality, sex and its consequences, appropriate and inappropriate behaviours, relationships, keeping safe, assertiveness skills, social skills, positive self‐esteem, emotional vocabulary, improvement in attitudes towards sexuality topics (see Table 7). The most reported outcomes across the studies were understanding of private body parts for males and females and understanding of what contraception is, both reported in 13 studies. Out of 92 outcomes, 78.2% (72) were knowledge‐based (e.g. understanding of what sexual intercourse is), 8.6% (8) of outcomes were skills based (e.g. social skills, assertiveness skills), 9.7% (9) of outcomes were about attitudes and feelings (e.g. positive self‐esteem, improvement in attitudes towards homosexuality) and 3.5% (3) of outcomes were behaviour‐based (e.g. engagement in inappropriate sexual behaviour).

**Table 7:** RSE outcomes reported for students in further education (16–25 years)

Outcome domain	Outcome	Frequency of reporting and study reference	Who reported/measured
Understanding of human body	Understanding of private body parts for males and females	13 studies: Finlay *et al*. (2015); Gokgoz *et al*. (2021); Kammes (2019); Pownall *et al*. (2012); Williamson (2017); Löfgren‐Mårtenson (2012); Dukes and McGuire (2009) ; Graff *et al*. (2018); Garwood and McCabe (2000); Plaks *et al*. (2010); Box and Shawe (2014); Kostigen (2020); Luque and Lugo (2015)	Teachers and school staff, parents, students with ID, measured by researchers
	Understanding of puberty related body changes	4 studies: Pownall *et al*. (2012); Box and Shawe (2014); Karellou (2017); Luque and Lugo (2015)	Parents, students with ID and content of RSE
	Understanding of what wet dreams are	3 studies: Frawley and Wilson (2016); Box and Shawe (2014); Peters (2007)	Students with ID, teachers
	Understanding of what self‐examination is for cancer screening (includes checking private body parts for cancer, e.g. breasts)	1 study: Box and Shawe (2014)	Content of RSE
Understanding of hygiene	Understanding of personal hygiene for males and females	4 studies: Nelson *et al*. (2020); Phasha and Runo (2017); Wiseman and Ferrie (2020); Graff *et al*. (2018)	Teachers, students with ID
	Hygiene skills	1 study: Kostigen (2020)	School staff
	Understanding of sexual hygiene (e.g. knowing to clean yourself after masturbation)	1 study; Kammes (2019)	Parents
Understanding of menstruation	Understanding of what menstruation is^†^	6 studies: Gokgoz *et al*. (2021); Graff *et al*. (2018); Box and Shawe (2014); Wiseman and Ferrie (2020); Garwood and McCabe (2000); Peters (2007)	Parents, teachers, students with ID, and measured by researchers
	Understanding physiology of menstruation	1 study: Bleazard (2010)	Students with ID
	Understanding of how to manage menstruation pain	1 study: Wiseman and Ferrie (2020)	Students with ID
	Understanding of how to identify problems with menstruation	1 study: Wiseman and Ferrie (2020)	Students with ID
	Understanding of how menstruation cycle is linked to pregnancy	1 study: Wiseman and Ferrie (2020)	Students with ID
	Understanding of how long the menstruation last over the life course and what the end of menstruation means	2 studies: Wiseman and Ferrie (2020); Box and Shawe (2014)	Students with ID and content of RSE
	Understanding of how to choose, use menstrual products and the right times to change them	2 studies: Phasha and Runo (2017); Wiseman and Ferrie (2020)	Teachers, students with ID
	Knowing how to identify changes in menstruation and possible issues that warrant medical attention	1 study: Wiseman and Ferrie (2020)	Students with ID
Understanding of human sexuality	Able to identify own gender	1 study: Dukes and McGuire (2009)	Measured by researchers
	Understanding of what different sexualities are (homosexuality, bisexuality, and heterosexuality)	3 studies: Pownall *et al*. (2012); Garwood and McCabe (2000); Peters (2007)	Parents, teachers and measured by researchers
Understanding sex and its consequences	Understanding of what sexual intercourse is^‡^	7 studies: Nelson *et al*. (2020); Bleazard (2010); Garwood and McCabe (2000); Karellou (2017); Peters (2007); Ustilaitė and Petrauskienė (2018); Luque and Lugo (2015)	Teachers, parents, students with ID and measured by researchers
	Knowledge of sexual behaviour (includes things you do by yourself and things you do with others)	2 studies: Plaks *et al*. (2010); Box and Shawe (2014)	Measured by researchers
	Understanding of what homosexual sexual experiences are	2 studies: Löfgren‐Mårtenson (2012); Box and Shawe (2014)	Students with ID and measured by researchers
	Understanding of what happens when men and women have an orgasm	1 study: Garwood and McCabe (2000)	Measured by researchers
	Understanding of how to have sex	2 studies: Phasha and Runo (2017); Frawley and Wilson (2016)	Students with ID
	Understanding of how to enjoy sex	1 study: Phasha and Runo (2017)	Students with ID
	Understanding of what contraception is (including different options of contraception, its side effects, that sometimes contraception does not work, e.g. condoms can break, how to use it and how it fits in with family planning for the future)	13 studies: Phasha and Runo (2017); Kammes (2019); Pownall *et al*. (2012); Löfgren‐Mårtenson (2012); Wiseman and Ferrie (2020); Graff *et al*. (2018); Frawley and Wilson (2016); Box and Shawe (2014); Garwood and McCabe (2000); Karellou (2017); Lafferty *et al*. (2012); Peters (2007); Luque and Lugo (2015)	Students with ID, parents, teachers and measured by researchers
	Understanding what STDs are	8 studies: Nelson *et al*. (2020); Phasha and Runo (2017); Pownall *et al*. (2012); Löfgren‐Mårtenson (2012); Williams *et al*. (2014); Wiseman and Ferrie (2020); Lafferty *et al*. (2012); Wilkenfeld and Ballan (2011)	Teachers, parents, students with ID
	Understanding consequences of sexual activity	4 studies: Williamson (2017); Plaks *et al*. (2010); Ustilaitė and Petrauskienė (2018); Wilkenfeld and Ballan (2011)	Students with ID, parents and measured by researchers
	Understanding consequences of having multiple sexual partners or ‘one‐night stand’ (e.g. STDs)	2 studies: Phasha and Runo (2017); Williamson (2017)	Teachers, students with ID
	Understanding of what pregnancy is	8 studies: Nelson *et al*. (2020); Phasha and Runo (2017); Löfgren‐Mårtenson (2012); Pownall *et al*. (2011); Garwood and McCabe (2000); Lafferty *et al*. (2012); Ustilaitė and Petrauskienė (2018); Wilkenfeld and Ballan (2011)	Teachers, students with ID, parents and measured by researchers
	Understanding that is hard to be a parent when you are young	1 study: Löfgren‐Mårtenson (2012)	Students with ID
	Understanding of how to take care of baby	2 studies: Graff *et al*. (2018); Box and Shawe (2014)	Students with ID and measured by researchers
	Understanding of how child develops	1 study: Williamson (2017)	Students with ID
	Understanding what abortion is	1 study: Garwood and McCabe (2000)	Measured by researchers
	Understanding what masturbation is^§^	5 studies: Pownall *et al*. (2012); Box and Shawe (2014); Garwood and McCabe (2000); Lafferty *et al*. (2012); Luque and Lugo (2015)	Parents, teachers and measured by researchers
	Understanding what kiss is and why people kiss	2 studies: Box and Shawe (2014); Ustilaitė and Petrauskienė (2018)	Parents and measured by researchers
Understanding appropriate and inappropriate behaviours	Understanding of appropriate and inappropriate sexual behaviours	5 studies: Finlay *et al*. (2015); Pownall *et al*. (2011); Pownall *et al*. (2012); Pryde and Jahoda (2018); Karellou (2017)	Teachers, parents, students with ID
	Understanding of appropriate ways to express sexual needs and desires	1 study: Phasha and Runo (2017)	Teachers
	Understanding the differences between public and private places (e.g. where you can be naked and where you cannot be naked)	4 studies: Pryde and Jahoda (2018); Graff *et al*. (2018); Box and Shawe (2014); Luque and Lugo (2015)	Parents, measured by researchers, and content of RSE
	Understanding of appropriate and inappropriate places for sexual activity (e.g. appropriate places to masturbate and not appropriate places to masturbate)	1 study: Pryde and Jahoda (2018)	Parents
	Not engaging in inappropriate sexual behaviour (actual behaviour was measured)	1 study: Dukes and McGuire (2009) ; Plaks *et al*. (2010)	Measured by researchers and content of RSE
	Understanding a concept of privacy	1 study: Kostigen (2020)	School staff
	Understanding of appropriate social behaviour	2 studies: Menon and Sivakami (2019); Wilkenfeld and Ballan (2011)	Parents, teachers
Understanding of relationships	Understanding of different relationships (e.g. friends, family, romantic relationships)	7 studies: Nelson *et al*. (2020); Phasha and Runo (2017); Löfgren‐Mårtenson (2012); Graff *et al*. (2018); Box and Shawe (2014); Graff *et al*. (2018); Luque and Lugo (2015)	Teachers, students with ID and measured by researchers
	Understanding of a concept of a friend	2 studies: Plaks *et al*. (2010); Garwood and McCabe (2000)	Measured by researchers
	Understanding of what marriage is	4 studies: Williamson (2018); Box and Shawe (2014); Garwood and McCabe (2000); Peters (2007)	Students with ID, teachers and measured by researchers
	Understanding of what is like being with someone in romantic relationship (including what is like living with a partner)	2 studies: Löfgren‐Mårtenson (2012); Nelson *et al*. (2020)	Students with ID, teachers
	Knowing how to flirt	1 study: Löfgren‐Mårtenson (2012)	Students with ID
	Understanding of how to express affection in different relationships and contexts	1 study: Luque and Lugo (2015)	Measured by researchers
	Able to identify abusive and healthy relationships	2 studies: Phasha and Runo (2017); Williamson (2017)	Teachers, students with ID
	Understanding of what dating is	2 studies: Pownall *et al*. (2012); Garwood and McCabe (2000)	Parents and measured by researchers
	Understanding of what attraction is	2 studies: Pownall *et al*. (2012); Box and Shawe (2014)	Parents, measured by researchers
	Understanding of what love is	3 studies: Löfgren‐Mårtenson (2012); Peters (2007); Ustilaitė and Petrauskienė (2018)	Students with ID, teachers, parents
	Understanding of what sexual relationships are	1 study: Phasha and Runo (2017)	Teachers
	Understanding how to have a relationship with a girlfriend/boyfriend	1 study: Frawley and Wilson (2016)	Students with ID
	Ability to make choices in relationships	1 study: Williams *et al*. (2014)	Students with ID
	Understanding of what girls/boys want in relationships	1 study: Frawley and Wilson (2016)	Students with ID
	Understanding of how to keep romantic relationships long‐term	1 study: Frawley and Wilson (2016)	Students with ID
	Understanding of how to break up in romantic relationships	1 study: Löfgren‐Mårtenson (2012)	Students with ID
	Understanding of what commitment in relationships means	1 study: Williamson (2017)	Students with ID
	Understanding of boundaries in relationships	1 study: Williamson (2017)	Students with ID
	Understanding what consent is	2 studies: Graff *et al*. (2018); Box and Shawe (2014)	Measured by researchers
	Understanding of consent in relationships (including how it is related to their current relationships)	1 study: Williamson (2017)	Students with ID
	Understanding of what peer pressure is	1 study: Pownall *et al*. (2012)	Parents
	Respecting yourself in romantic relationships (not sleeping with everyone)	1 study: Ustilaitė and Petrauskienė (2018)	Parents
Understanding of how to keep safe	Safety skills (keeping distance with strangers, not accepting gifts, money, or car rides from strangers, reporting when strangers call them)	8 studies: Finlay *et al*. (2015); Gokgoz *et al*. (2021); Kammes (2019); Pownall *et al*. (2012); Pryde and Jahoda (2018); Dukes and McGuire (2009) ; Plaks *et al*. (2010); Karellou (2017)	Teachers, parents, students with ID and measured by researchers
	Understanding of appropriate and inappropriate touch	3 studies: Pryde and Jahoda (2018); Box and Shawe (2014); Luque and Lugo (2015)	Parents, and content of RSE
	Understanding of what people are allowed to see you naked	2 studies: Graff *et al*. (2018); Kostigen (2020)	School staff and measured by researchers
	Understanding of what rape is	1 study: Phasha and Runo (2017)	Students with ID
	Understanding what to do in case of abuse	2 studies: Phasha and Runo (2017); Luque and Lugo (2015)	Students with ID, content of RSE
	Understanding of what sexual abuse is	3 studies: Bleazard (2010); Pownall *et al*. (2011); Box and Shawe (2014)	Parents and content of RSE
	Understanding of how to protect yourself from sexual abuse	1 study: Peters (2007)	Teachers
	Understanding of different types of abuse	2 studies: Graff *et al*. (2018); Box and Shawe (2014)	Students with ID and content of RSE
	Knowing the risks of being on the internet	1 study: Löfgren‐Mårtenson (2012)	Students with ID
Assertiveness skills	Able to communicate (with behaviour, e.g. pushing) or say ‘no’ to people if they (students) do not agree with something or do not want to participate in activity	5 studies: Box and Shawe (2014); Finlay *et al*. (2015); Dukes and McGuire (2009) ; Plaks *et al*. (2010); Luque and Lugo (2015)	Teachers, students with ID and content of RSE
Social skills	Social skills	3 studies: Nelson *et al*. (2020); Pownall *et al*. (2012); Plaks *et al*. (2010)	Teachers, parents, and content of RSE
	Listening to and respecting your peers	1 study: Luque and Lugo (2015)	Measured by researchers
	Respecting other peoples private space	1 study: Luque and Lugo (2015), Kostigen (2020)	School staff, measured by researchers
	Understanding of what a term ‘empathy’ means	1 study: Graff *et al*. (2018)	Measured by researchers
	How to communicate, socialise with opposite sex (e.g. what is appropriate for boys to say to girls and what is not appropriate)	1 study: Frawley and Wilson (2016); Ustilaitė and Petrauskienė (2018)	Students with ID, parents
	How to deal with other people (e.g. people you like or do not like)	1 study: Williamson (2017)	Students with ID
	Respecting peoples differences and different opinions	1 study: Williamson (2017)	Students with ID
Positive self‐esteem	Understanding of what is like being a woman or a man with disability	1 study: Williamson (2017)	Students with ID
	Loving and accepting yourself	2 studies: Williamson (2017); Plaks *et al*. (2010)	Students with ID and content of RSE
	Being comfortable and confident in yourself	2 studies: Williamson (2017); Luque and Lugo (2015)	Students with ID, content of RSE
	Understanding of what a term “self‐esteem” means	1 study: Graff *et al*. (2018)	Measured by researchers
Emotional vocabulary	Ability to recognise and express emotions	1 study: Luque and Lugo (2015); Lafferty *et al*. (2012)	Teachers and content of RSE
Improvement in attitudes towards sexuality topics	Improvement in attitudes towards friendships	1 study: Garwood and McCabe (2000)	Measured by researchers
	Improvement in attitudes towards dating and intimacy	1 study: Garwood and McCabe (2000)	Measured by researchers
	Improvement in attitudes towards sexual interaction	1 study: Garwood and McCabe (2000)	Measured by researchers
	Improvement in attitudes towards masturbation	1 study: Garwood and McCabe (2000)	Measured by researchers
	Improvement in attitudes towards homosexuality	1 study: Garwood and McCabe (2000)	Measured by researchers

STDs, sexually transmitted diseases; ID, intellectual disability.

^†^ One study contradict that this should be an outcome of RSE (Goli *et al*. 2020).

^‡^ One study contradicts that this should be an outcome of RSE (Menon and Sivakami 2019).

^§^ One study contradict that this should be an outcome of RSE (Menon and Sivakami 2019).

Only one of 37 studies (Graff *et al*. 2018) reported how some of the extracted outcomes were measured. In this study, students' understanding of what consent is, public and private places, terms such as ‘self‐esteem’ and ‘empathy’ were assessed by asking students to match a word (e.g. consent) with a correct definition out of several possible definitions on a paper questionnaire developed for the study. Understanding of private body parts for males and females was assessed by asking students to draw an ‘X’ on private parts on pictures of male and female bodies. Five studies (Garwood & McCabe 2000; Dukes & McGuire 2009; Plaks *et al*. 2010; Box & Shawe 2014) only mentioned the names of the instruments used to evaluate the outcomes. For example, in the Dukes and McGuire (2009) study, researchers administered the Sexual Consent and Education Assessment (SCEA) (Kennedy 1993), an interview schedule, to students with intellectual disability to measure students' understanding of sexuality topics (e.g. private body parts, ability to identify own gender). Students' engagement in inappropriate sexual behaviour and understanding of safety practices was assessed by administering the SCEA instrument to students' carers. However, authors did not report specific questions students and carers might have been asked.

In Luque and Lugo's (2015) study, students' understanding of private body parts, how to express affection in different relationships, and ability to listen and respect your peers was assessed based on students' reports (e.g. whether they think they are able respect their peers), teacher's and parents' reports of student's behaviour. However, what specific behaviours teachers and parents were considering when evaluating these outcomes nor how students' understanding of private body parts was assessed (e.g. not showing private body parts in public or asking students to describe it verbally) were not specified in the paper.

### Outcomes reported by parents, teachers, and students in further education

There was an overlap between 14 (31%) out of 44 RSE outcomes reported by parents and teachers for students with ID in further education. These outcomes relate to private body parts, understanding of sex and its consequences, relationships, different sexualities, appropriate and inappropriate behaviours, social skills and how to keep safe. We also looked at whether outcomes that were reported only by parents and only by teachers were different. We found that outcomes identified by each stakeholder group separately tended to be similar to outcomes reported by both stakeholder groups together, suggesting that RSE outcomes reported by teachers and parents tend to converge. For example, parents reported an outcome of RSE ‘understanding of what menstruation is’, whereas teachers reported an outcome of RSE ‘understanding of how to choose, use menstrual products’. It is likely that when parents discuss what menstruation is with their children, parents also discuss how to choose menstrual products, but this information was not specified in the papers. However, in two studies (Menon and Sivakami 2019; Goli *et al*. 2020) parents and teachers did not agree that understanding of menstruation, masturbation, and sexual intercourse should be an outcome of RSE for students with intellectual disability.

Students with intellectual disability reported 52 individual outcomes of RSE that they perceive as important. The most frequently reported outcomes of RSE by students with intellectual disability were understanding of contraception and what sexually transmitted diseases are, both reported in four studies (Löfgren‐Mårtenson 2012; Williams *et al*. 2014; Phasha and Runo 2017; Wiseman and Ferrie 2020). However, students with intellectual disability reported different outcomes as meaningful which were not mentioned by other stakeholders in the studies. Students reported that in RSE they would like to learn how to identify problems with menstruation, how to have sex and enjoy it, how to use contraception (e.g. how to apply condoms), how to take care of a baby, what are homosexual sexual experiences, what it is like living with a partner, how to flirt, how to break up in relationships, how to love and accept yourself, and what to do in case of abuse. On the other hand, outcomes reported by parents and teachers were about how to protect yourself and appropriate and inappropriate behaviours.

### Characteristics of the outcome measurement instruments

Five instruments were used to measure RSE outcomes in the retrieved studies (see Table 8).

**Table 8:** Characteristics of the outcome measurement instruments

Where located	Instrument
Box and Shawe (2014)	**Name:** “Not a Child Anymore” (Fraser 1987). **Constructs:** Knowledge of social behaviour: kissing, sexual assault, marriage and caring for children. Knowledge of sexual topics: masturbation, menstruation, and contraception. **Target population:** not reported. **Intended contexts of use:** not reported. **Mode of administration:** self‐report questionnaire administered to people with ID. **When administered:** before and after program (specific timings not reported). **No. of items and subscales:** 111 questions over 16 sections (which are not specified). **Response options:** not reported. **Original language:** not reported. **Available translations:** not reported
Dukes and McGuire (2009)	**Name:** The Sexual Consent and Education Assessment (SCEA) (Kennedy 1993) **Constructs:** knowledge of safety practices; knowledge of human sexuality that contained: knowledge of the physical self, knowledge of sexual functioning, knowledge of choices and consequences in sexual activities; inappropriate sexual behaviour. **Target population:** cognitively impaired individuals (ID and traumatic brain damage). **Intended contexts of use:** not reported. **Mode of administration:** interview based. The knowledge of human sexuality scale delivered in 1 interview (of 20–60 min) with the person with cognitive impairment and the knowledge of safety practices and inappropriate behaviour scales delivered in separate interviews (of 15–25 min each) with a caregiver. **When administered:** pre intervention, after each session and at 6 months follow‐up. **No. of items and subscales:** 35‐item instrument with three scales: Knowledge of Human Sexuality (K‐Scale) (only first 4 questions were administered), Safety Practices (S‐Scale) and Inappropriate Sexual Behaviour Scale. **Response options:** Questions in the Knowledge and Safety Practices scale are scored as 0 (fail) and 1 (pass). Questions in the Inappropriate Sexual Behaviour scale are scored on 5‐point Likert scale (ranging from 1 to 5) with responses “never, rarely, occasionally, frequently, almost always”. **Original language:** English. **Available translations:** not reported.
Garwood and McCabe (2000); Plaks *et al*. (2010)	**Name:** The Sexuality Knowledge, Experience, Feelings and Needs Scale for people with Intellectual Disability (Sex Ken‐ID) (McCabe 1994). **Constructs:** Knowledge of sexual topics: friendship, dating and intimacy, marriage, body part identification, sex education, menstruation, sexual interaction, contraception, pregnancy, abortion and childbirth, STDs, masturbation, and homosexuality. Feelings towards sexuality topics: friendship, dating and intimacy, marriage, body part identification, sex education, menstruation, sexual interaction, contraception, pregnancy, abortion and childbirth, STDs, masturbation, and homosexuality. **Target population:** people with mild ID. **Intended contexts of use:** not reported. **Mode of administration:** interviews with people with ID (3 separate interviews lasting of 1–1.5 h). **When administered:** before and after program (specific timings not reported). **No. of items and subscales:** 248 item interview schedule classified into Knowledge, Experience, Feelings and Needs areas (only knowledge and feelings scales used in the study). **Response options:** The knowledge questions are open‐ended questions, with responses scored as 0, 1 or 2. The feelings questions are the yes‐no type (responses scored as 1 to 2) or scored on a 5‐point Likert scale (ranging from 1 to 5): “very bad, bad, neutral, good, very good”. 2. A total score is obtained for each of the sub‐areas (knowledge or feelings) within each subscale (e.g. friendship, STDs). **Original language:** English. **Available translations:** not reported.
Plaks *et al*. (2010)	**Name:** The Skills and Independent Activities Questionnaire (Argaman 2003). **Constructs:** social skills and engagement in independent activities at home. **Target population:** not reported. **Intended contexts of use:** not reported. **Mode of administration:** parent‐report questionnaire. **When administered:** pre‐post education. **No. of items and subscales:** 19 items “Social Entertainment” (9 items) and “Independent Activities” (10 items). **Response options:** not reported. **Original language:** not reported. **Available translations:** not reported.
Plaks *et al*. (2010)	**Name:** The assessment of the understanding of the concept of a friend (Argaman 2003). **Constructs:** not reported. **Target population:** not reported. **Intended contexts of use:** not reported. **Mode of administration:** self‐report questionnaire administered to people with ID. **When administered:** pre‐post education. **No. of items and subscales:** not reported. **Response options:** not reported. **Original language:** not reported. **Available translations:** not reported.

ID, intellectual disability.

The Not a Child Anymore (Fraser 1987) instrument was administered in Box and Shawe's (2014) study with people with intellectual disability aged 20–24 years to assess their knowledge of sexual behaviour (e.g. kissing and sexual assault) and knowledge of sexual topics (e.g. masturbation and contraception). The authors reported that this is a self‐report questionnaire that contains 111 questions, and no other information about the instrument was provided (e.g. whether it was developed for people with intellectual disability or measurement properties).

The Sexual Consent and Education Assessment (SCEA) (Kennedy 1993) is an interview schedule developed to assess individuals' with cognitive impairment (e.g. intellectual disability or traumatic brain injury) capacity to consent to sexual activities and identify areas where individuals need further education. This instrument was administered in Dukes and McGuire's (2009) study before the RSE delivery, at the end of each session and at 6 months' follow‐up to students with intellectual disability aged 22–23 years. It contained 35 questions distributed over three scales: Knowledge of Human Sexuality Scale (only the first 4 items of the scale were delivered to students in an interview lasting 20–50 min), Safety Practices Scale, and Inappropriate Sexual Behaviour Scale (the former two scales delivered in a separate interview to students' carers). Dukes and McGuire (2009) reported that the instrument ‘has high internal stability and inter‐rater reliability and satisfactory test–retest reliability’ without providing data to support these statements.

The Sexuality Knowledge, Experience, Feelings and Needs Scale for people with Intellectual Disability (Sex Ken‐ID) (McCabe 1994) is an interview schedule developed for people with mild intellectual disability to assess their knowledge, experiences, feelings and needs over 12 sexuality topics: Friendship, Dating and Intimacy, Marriage, Body Part Identification, Sex and Sex Education, Menstruation, Sexual Interaction, Contraception, Pregnancy, Abortion and Childbirth, Sexually Transmitted Diseases, Masturbation, and Homosexuality. The instrument contains 248 questions administered in three separate interviews each lasting 1–1.5 h. This instrument was administered in two studies (Garwood and McCabe 2000; Plaks *et al*. 2010) to students with intellectual disability aged 12–25 years before and after RSE delivery. In Garwood and McCabe (2000) only the Knowledge and Feelings scales were administered. Authors reported that the instrument has ‘moderate to high’ internal consistency within each of 12 sexuality topics. In their paper, authors provided Cronbach's alpha for selected scales: 0.47 for Knowledge of Dating and Intimacy; 0.79 for the Feelings of Dating and Intimacy scale; 0.79 for Knowledge of Sexual Interaction scale and 0.46 for Feelings of Sexual Interaction scale. The authors reported that the feelings scale was less reliable as it has only 2–3 questions within each of 12 sexuality topics.

The Skills and Independent Activities Questionnaire (Argaman 2003), a parent‐report scale, was administered in Plaks *et al*. (2010) to assess social skills and engagement in independent activities at home before and after RSE delivery for students with intellectual disability aged 15–25 years. The assessment of the understanding of the concept of a friend (Argaman 2003), a self‐report questionnaire, was also administered in Plaks *et al*. (2010) to students with intellectual disability. No other information about these two instruments was provided in the paper (e.g. whether the instruments were developed for people with intellectual disability and instruments' measurement properties).

### Second stage of the review

A separate systematic search was conducted for each of the five instruments: the Not a Child Anymore (Fraser 1987); the SCEA (Kennedy 1993); the Sex Ken‐ID (McCabe 1994); the Skills and Independent Activities Questionnaire (Argaman 2003) and the assessment of the understanding of the concept of a friend (Argaman 2003). For three instruments‐ the Not a Child Anymore (Fraser 1987), the SCEA (Kennedy 1993) and the Sex Ken‐ID (McCabe 1994) ‐ the searches retrieved studies additional to the ones retrieved in the first stage of the review (see supporting information for PRISMA flow diagrams).

The search of studies on the SCEA (Kennedy 1993) retrieved eight individual articles which were screened on title and abstract. Four papers were screened on full text, but they did not meet the inclusion criteria: one did not evaluate measurement properties of the instrument; one was a duplicate and two provided information on the instrument's development, internal consistency, criterion validity and construct validity, but evaluations were carried out involving people with intellectual disability aged above 25 years.

The search on the Sex Ken‐ID (McCabe 1994) retrieved six individual studies which were screened on title and abstract. Three studies were screened on full‐text, but they did not meet the inclusion criteria: two did not evaluate the instrument and one provided information on the instrument's development, internal consistency, and test–retest reliability, but evaluations were carried out with people with intellectual disability aged above 25 years.

The search on the Not a Child Anymore (Fraser 1987) retrieved two individual articles which were screened on title and abstract and full‐text, but these studies did not meet the inclusion criteria: one did not evaluate the instrument and one full‐text was not available.

## Discussion

This review identified 135 RSE outcomes for students with intellectual disability aged 5–25 years that were reported as important by stakeholders across 42 studies. The majority of outcomes (92) related to students with intellectual disability in further education (aged 16–25 years), 43 outcomes were extracted for students in secondary education (aged 11–16 years) and there were no outcomes reported for students with intellectual disability in primary education (aged 5–10 years).

Outcomes were predominantly knowledge based and focused on improving understandings of the human body, hygiene, relationships, sexual intercourse and its consequences, how to protect yourself from potential abuse, and appropriate and inappropriate behaviours rather than pertaining to skills and attitudes. However, many students with intellectual disability have difficulties understanding abstract and complex sexuality concepts (e.g. consent in relationships) and applying the new knowledge in practice (Finlay *et al*. 2015; Bundock & Hewitt 2017). This also possibly explains why students with intellectual disability in the retrieved studies reported that in RSE lessons they want to develop skills (e.g. how to apply condoms) to learn how to have romantic relationships, enjoy sex, embrace their sexuality. In contrast, outcomes reported by the parents and teachers focused on developing knowledge of appropriate and inappropriate behaviours and how to keep safe.

Most of the outcomes were extracted from qualitative data and only eight studies reported outcomes measured following RSE delivery. However, RSE evaluation studies included in the present review were reported in ways that suggest they were not developed systematically; the aim of RSE programmes was rarely specified, none of the studies mentioned theoretical underpinnings of their programmes and only two studies delivered RSE based on materials developed for people with intellectual disability. Studies also lacked detail on what specific outcomes were measured, why these outcomes were selected and how the measurement was performed.

These represent some significant limitations as RSE programmes without clearly specified aims and theoretically linked outcomes cannot be robustly evaluated to determine the effects of their implementation (Fernandez *et al*. 2019). Without a theory of change, an understanding of what ‘active ingredients’ may bring about change is also hindered (O'Cathain *et al*. 2019). Conclusions drawn from such studies are of limited value and it is very challenging to replicate the findings in different settings without additional information.

In addition, RSE programme aims should have been developed based on needs assessment and stakeholders' views (Fernandez *et al*. 2019; Skivington *et al*. 2021). However, we found that none of the studies reported including the views of students with intellectual disability in the development of RSE programmes. This is a significant limitation as learner‐centred teaching that incorporates students' interests and individual needs is associated with higher students' motivation, achievement, and skills development (Meece *et al*. 2003; Alfassi 2004; Dano‐Hinosolango and Vedua‐Dinagsao 2014). Thus, these RSE programmes are likely to be less relevant and effective for students with intellectual disability.

RSE content delivered to students with intellectual disability was only briefly mentioned in the papers and focused mainly on protection and managing risks. Compared with the suggested topics of comprehensive sexuality education by the World Health Organization (2010), the content of RSE in the studies did not seem to include topics on sexual identity, sexual pleasure, how to seek help and information on sexuality topics (e.g. how to access sexual health services), how to express needs and wishes within relationships and information on their sexual rights (including the right to marriage and choosing romantic partners). This gap also contrasts with the RSE statutory guidance for English schools (DfE 2019).

Our review identified that no instruments have been validated to measure outcomes of RSE for students with intellectual disability aged 5–25 years. Searches carried out on measurement properties of the five identified instruments revealed that only two instruments (the Sex Ken‐ID and SCEA) had been evaluated. However, the evaluations had not been performed involving students with intellectual disability aged 5–25 years. For example, the Sex Ken‐ID instrument was administered in 2 studies (Garwood and McCabe 2000; Plaks *et al*. 2010) to students with intellectual disability aged 12–25 years, but the piloting of the instrument was performed with people with intellectual disability aged above 27 years (McCabe *et al*. 1999). Further, the measure's reported internal reliability was low (e.g. Cronbach's alpha for Knowledge of Dating and Intimacy and Feelings of Sexual Interaction scales are below 0.60) (Garwood and McCabe 2000) and we found no studies on its content validity.

Neither instrument was designed to be used across the intellectual disability spectrum, and their content is likely outdated (both developed in the 1990s). The SCEA instrument was designed for people with cognitive impairments such as people with traumatic brain injury and people with intellectual disability. However, those two populations are not the same and the instrument might fail to capture relevant aspects for people with intellectual disability. The administration of the Sex Ken‐ID requires three individual interviews of 1–1.5 h and is not feasible with students more severe intellectual disability and verbal communication difficulties. Therefore, additional studies are needed to evaluate these instruments' validity, reliability and feasibility with students with intellectual disability.

### Strengths and limitations

To our knowledge, this is the first systematic review on RSE outcomes for students with intellectual disability. This is also the first systematic review with the aim of evaluating RSE outcome measurement instruments' measurement properties. We used a comprehensive search strategy that involved searching nine electronic databases and grey literature and including studies published in any language. Thus, we were able to capture a range of stakeholders' perspectives of meaningful outcomes.

Although the review included studies from several countries, the majority of studies were carried out in Western countries. Therefore, the identified list of outcomes might be less generalisable to non‐Western countries.

Outcomes identified this review also do not represent RSE outcomes for all students with intellectual disability. Our review identified that there were no RSE outcomes reported specifically for students with intellectual disability in primary education (5–10 years). It is likely that RSE outcomes for younger students would differ from the outcomes extracted for older students with intellectual disability. Further, outcomes reported by students with intellectual disability were extracted from studies that carried out interviews with verbal students. There were no studies that had included the views of students with more severe intellectual disability and verbal communication difficulties.

We were not able to retrieve full‐texts of 23 papers (7%) to see if they would meet our inclusion criteria and there is the possibility that we missed some RSE outcomes.

### Implications for research and practice

Our review identified that there is a discrepancy of RSE outcomes perceived as important between students with intellectual disability and their parents and teachers. Therefore, it is essential to include students with intellectual disability in the development of RSE. Our findings suggest a need for RSE programmes to focus on skills development rather than solely providing facts for this population. This could be carried out by including activities that allow students with intellectual disability to practise skills (e.g. using role plays in schools or asking students to complete ethically sensitive activities such as applying condoms at home) and embedding the new skills learned in everyday life (e.g. asking a student's consent before touching a student).

RSE programme developers could consult available guidelines more closely such as the Information Mapping toolkit (Fernandez *et al*. 2019) or the UK Medical Research Council guidance on developing complex interventions (Skivington *et al*. 2021) to ensure that programmes developed are comprehensive and reflect the needs and priorities of students with intellectual disability. Findings here clearly highlighted the need to develop validated instruments to assess the effectiveness of RSE with students with different levels of intellectual disability and verbal abilities in school settings.

The list of outcomes resulting from the current review could be used to guide research and school‐based evaluations in Western countries. This list of outcomes could also be used as a starting point for researchers interested in developing a stakeholder consensus‐based COS of RSE for students with intellectual disability in different cultural contexts.

## Source of funding

This work is supported by the Economic and Social Research Council PhD studentship awarded to LP: ES/P000592/1. The funder had no role in the design of the study, writing the manuscript, and the decision to submit the article for publication.

## Conflict of interest

The authors declare that they have no conflicts of interest.

## Ethics approval statement

Ethical approval for this systematic review was not obtained as it does not involve primary data collection from participants.

## Supporting information


**Table S1:** Example of search strategy used in the 1st and 2nd stage of review
**Table S2:** RSE outcomes for students with intellectual disability that age was not specified
**Figure S1:** PRISMA Flow Diagram: study selection process on the Sexual Consent and Education Assessment (SCEA)
**Figure S2:** PRISMA Flow Diagram: study selection process on the Sexuality Knowledge, Experience, Feelings and Needs Scale for people with Intellectual Disability (Sex Ken‐ID)
**Figure S3:** PRISMA Flow Diagram: study selection process on the Not a Child Anymore

## Data Availability

Data sharing is not applicable to this article as no new data were created or analysed in this study.
