# Supplementary material for: A systematic review of relationships and sex education outcomes for students with intellectual disability reported in the international literature
Source: J Intellect Disabil Res. 2022 Jun 13;66(7):577–616. doi: 10.1111/jir.12952 (PMC9328360; doi:10.1111/jir.12952)

# Supplementary material

## Supplementary material 1: Example of search strategy used in the 1st and 2nd stage of review

| **1^st^ stage of the review** | | |
| --- | --- | --- |
| **Medline (Ovid) searched on 31/04/2021** | | |
| **Set** | **Search Terms** | **Results** |
| 1 | Intellectual Disability/ | 55535 |
| 2 | Autistic Disorder/ | 20890 |
| 3 | Down Syndrome/ | 24701 |
| 4 | Smith-Magenis Syndrome/ | 202 |
| 5 | Rett Syndrome/ | 2645 |
| 6 | Lesch-Nyhan Syndrome/ | 1213 |
| 7 | Prader-Willi Syndrome/ | 2950 |
| 8 | Angelman Syndrome/ | 1219 |
| 9 | Fragile X Syndrome/ | 5164 |
| 10 | Cri-du-Chat Syndrome/ | 677 |
| 11 | De Lange Syndrome/ | 834 |
| 12 | Rubinstein-Taybi Syndrome/ | 512 |
| 13 | Developmental Disabilities/ | 20665 |
| 14 | Education, Special/ | 8870 |
| 15 | 1 or 2 or 3 or 4 or 5 or 6 or 7 or 8 or 9 or 10 or 11 or 12 or 13 or 14 | 134213 |
| 16 | ("learning disab*" or "learning difficult*" or "learning disorder" or "learning impair*" or "intellectual* disab*" or "intellectual* impair*" or "intellectual dysfunction" or "development* disab*" or "development* disorder*" or "development* impair*" or "intellectual developmental disorder" or "mental* deficien*”" or "mental* retard*" or "mental* handicap*" or "mental* disab*" or "mental insufficiency" or "mental* impair*" or "mental* challenged" or IQ or "subaverage intelligence" or "cognitive impair*" or autis* or ASD or Asperg* or "Autism Spectrum Disorders" or "Down* Syndrome" or "trisomy 21" or "Smith-Magenis" or Rett* or "Lesch-Nyhan" or "Prader-Willi" or Angelman or "fragile X" or "Cri-du-chat" or "Cornelia de Lange" or "de Lange" or "Rubinstein-Taybi" or "special education* need*" or SEN or "special education* need* and disab*" or SEND or "special need*" or "additional support need*" or ASN).mp. [mp=title, abstract, original title, name of substance word, subject heading word, floating sub-heading word, keyword heading word, organism supplementary concept word, protocol supplementary concept word, rare disease supplementary concept word, unique identifier, synonyms] | 410172 |
| 17 | 15 or 16 | 415360 |
| 18 | Sex Education/ | 8901 |
| 19 | Sexual Health/ed | 128 |
| 20 | Psychosexual Development/ | 3564 |
| 21 | Sexuality/px | 2111 |
| 22 | Sexual Behavior/ed, px | 11773 |
| 23 | ("sex* education" or "sex* curricul*" or "sex* and relationship* education" or "sex* and relationship* curricul*" or "relationship* and sex* education" or "relationship* and sex* curricul*" or "relationship* education" or "relationship* curricul*" or "sex* health education" or "sex* health curricul*").mp. [mp=title, abstract, original title, name of substance word, subject heading word, floating sub-heading word, keyword heading word, organism supplementary concept word, protocol supplementary concept word, rare disease supplementary concept word, unique identifier, synonyms] | 14591 |
| 24 | ("reproductive health education" or "reproductive health curricul*" or "life skills education" or "life skills curricul*" or "personal social health and economic education" or "personal social and health education" or PSHE or PSHEE or "social personal health education" or SPHE or "personal social health and citizenship education" or PSHCE or "personal social and emotional development" or PSED).mp. [mp=title, abstract, original title, name of substance word, subject heading word, floating sub-heading word, keyword heading word, organism supplementary concept word, protocol supplementary concept word, rare disease supplementary concept word, unique identifier, synonyms] | 493 |
| 25 | 18 or 19 or 20 or 21 or 22 or 23 or 24 | 30907 |
| 26 | (child* or youth* or pupil* or adolesc* or pre-adolesc* or pubert* or pediatric* or paediatric* or boy* or girl* or teen* or juvenil* or minor* or school* or student* or learner* or "young people*" or "young person*" or "young adult*" or "young women" or "young woman" or "young men" or "young man" or "early adult*" or "emerging adult*").mp. | 4861166 |
| 27 | 17 and 25 and 26 | 501 |
| 28 | limit 27 to humans | 465 |
| 29 | limit 28 to yr="1999 -Current" | **244** |

| **2^nd^ stage of the review** | | |
| --- | --- | --- |
| **Embase** <1980 to 2021 Week 31> | | |
| 1 | (“learning disab*” or “learning difficult*” or “learning disorder” or “learning impair*” or “intellectual* disab*” or “intellectual* impair*” or “intellectual dysfunction” or “development* disab*” or “development* disorder*” or “development* impair*” or “intellectual developmental disorder” or “mental* deficien*”” or “mental* retard*” or “mental* handicap*” or “mental* disab*” or “mental insufficiency” or “mental* impair*” or “mental* challenged” or IQ or “subaverage intelligence” or “cognitive impair*” or autis* or ASD or Asperg* or “Autism Spectrum Disorders” or “Down* Syndrome” or “trisomy 21” or “Smith-Magenis” or Rett* or “Lesch-Nyhan” or “Prader-Willi” or Angelman or “fragile X” or “Cri-du-chat” or “Cornelia de Lange” or “de Lange” or “Rubinstein-Taybi” or “special education* need*” or SEN or “special education* need* and disab*” or SEND or “special need*” or “additional support need*” or ASN).mp. [mp=title, abstract, heading word, drug trade name, original title, device manufacturer, drug manufacturer, device trade name, keyword, floating subheading word, candidate term word] | 567462 |
| 2 | Down Syndrome/ | 32076 |
| 3 | Cornelia De Lange Syndrome/ | 1232 |
| 4 | Crying Cat Syndrome/ | 600 |
| 5 | Fragile X Syndrome/ | 8972 |
| 6 | Rett Syndrome/ | 5542 |
| 7 | Prader Willi Syndrome/ | 5974 |
| 8 | Williams Syndrome/ | 2636 |
| 9 | Learning Disabilities/ | 30073 |
| 10 | Developmental Disabilities/ | 11600 |
| 11 | Special Education/ | 3044 |
| 12 | intellectual impairment/ | 30184 |
| 13 | autism/ | 70405 |
| 14 | learning disorder/ | 30073 |
| 15 | developmental delay/ | 10209 |
| 16 | mental deficiency/ | 46220 |
| 17 | 1 or 2 or 3 or 4 or 5 or 6 or 7 or 8 or 9 or 10 or 11 or 12 or 13 or 14 or 15 or 16 | 575716 |
| 18 | “Sexual Consent and Education Assessment”.mp. [mp=title, abstract, heading word, drug trade name, original title, device manufacturer, drug manufacturer, device trade name, keyword, floating subheading word, candidate term word] | 2 |
| 19 | SCEA.mp. [mp=title, abstract, heading word, drug trade name, original title, device manufacturer, drug manufacturer, device trade name, keyword, floating subheading word, candidate term word] | 40 |
| 20 | 18 or 19 | 40 |
| 21 | 17 and 20 | **3** |
| 22 | “Sex KEN-ID”.mp. [mp=title, abstract, heading word, drug trade name, original title, device manufacturer, drug manufacturer, device trade name, keyword, floating subheading word, candidate term word] | 2 |
| 23 | “Sexuality Knowledge Experience Feelings and Needs Scale for people with Intellectual Disability”.mp. [mp=title, abstract, heading word, drug trade name, original title, device manufacturer, drug manufacturer, device trade name, keyword, floating subheading word, candidate term word] | 0 |
| 24 | 22 or 23 | 2 |
| 25 | 17 AND 24 | **2** |
| 25 | “Not a child anymore”.mp. [mp=title, abstract, heading word, drug trade name, original title, device manufacturer, drug manufacturer, device trade name, keyword heading word, floating subheading word, candidate term word] | 2 |
| 26 | 25 AND 17 | **1** |

## Supplementary material 2: RSE outcomes for students with intellectual disability that age was not specified

| **Outcome domain** | **Outcomes** | **Frequency of reporting and study reference** | **Who reported** |
| --- | --- | --- | --- |
| Understanding of the human body | Understanding of physical changes in adolescence | 1 study; Girgin-Büyükbayraktar et al., (2017) | Teachers |
|  | Able to name private body parts for males and females | 1 study; Hanass-Hancock et al., (2018) | Teachers |
|  | Understanding of reproductive anatomy and physiology | 1 study; Howard-Barr (2005) | Teachers |
|  | Understanding of reproduction | 1 study; Howard-Barr (2005) | Teachers |
|  | Understanding what puberty is | 1 study; Howard-Barr (2005) | Teachers |
| Understanding of sex and its consequences | Understanding of what sexual experiences are | 2 studies; Hanass-Hancock et al., (2018); Howard-Barr (2005) | Teachers |
|  | Understanding that sex can lead to pregnancy | 1 study; Aderemi (2014) | Teachers |
|  | Understanding what contraception is | 1 study; Howard-Barr (2005) | Teachers |
|  | Understanding what abortion is | 1 study; Howard-Barr (2005) | Teachers |
|  | Understanding of what STDs are | 1 study; Howard-Barr (2005) | Teachers |
|  | Understanding what HIV is | 1 study; Aderemi (2014) | Teachers |
|  | Satisfying sexual needs in an appropriate environment | 1 study; Girgin-Büyükbayraktar et al., (2017) | Teachers |
|  | Understanding what masturbation is | 1 study; Howard-Barr (2005) | Teachers |
|  | Masturbation in an appropriate environment | 1 study; Girgin-Büyükbayraktar et al., (2017) | Teachers |
|  | Understanding of what ejaculation is | 1 study; Howard-Barr (2005) | Teachers |
|  | Understanding of what sexual dysfunction is | 1 study; Howard-Barr (2005) | Teachers |
| Understanding of the human sexuality | Understanding gender roles | 1 study; Howard-Barr (2005) | Teachers |
|  | Understanding concept of illegal sexual activity | 1 study; Howard-Barr (2005) | Teachers |
|  | Understanding of different types of sexual orientations (e.g. bisexual, homosexual, heterosexual) | 1 study; Howard-Barr (2005) | Teachers |
| Understanding of appropriate and inappropriate behaviour | Understanding of appropriate and inappropriate touch (e.g. which regions they (students) must not touch of other people) | 1 study; Girgin-Büyükbayraktar et al., (2017) | Teachers |
| Understanding of hygiene | Understanding how to clean genital regions on their own | 1 study; Girgin-Büyükbayraktar et al., (2017) | Teachers |
| Understanding of how to stay safe | Understanding what body parts other people can touch and body parts other people cannot touch | 1 study; Girgin-Büyükbayraktar et al., (2017) | Teachers |
|  | Understanding of consensual or non-consensual touch | 1 study; Hanass-Hancock et al., (2018) | Teachers |
|  | Understanding of sexual abuse | 1 study; Howard-Barr (2005) | Teachers |
| Assertiveness skills | Able to say “no” if they don’t agree with something | 1 study; Hanass-Hancock et al., (2018) | Teachers |
|  | Able to show with their facial expressions if they do not agree with something | 1 study; Hanass-Hancock et al., (2018) | Teachers |
| Emotional vocabulary | Able to recognise and name different feelings | 1 study; Hanass-Hancock et al., (2018) | Teachers |
| Social skills | Social skills (no description provided) | 1 study; Hanass-Hancock et al., (2018) | Teachers |
|  | Social boundaries | 1 study; Hanass-Hancock et al., (2018) | Teachers |
| Understanding of relationships | Understanding of different types of relationships (e.g families, friendships, dating, marriage, and lifetime commitments) | 2 studies; Hanass-Hancock et al., (2018); Howard-Barr (2005) | Teachers |
|  | Understanding difference in ‘relationships’ between adults and learners or between girls and boys at school | 1 study; Hanass-Hancock et al., (2018) | Teachers |
|  | Understanding of appropriate relationships | 1 study; Hanass-Hancock et al., (2018) | Teachers |
|  | Understanding what love means | 2 studies; Hanass-Hancock et al., (2018) | Teachers |
| Tolerance towards other sexualities | Knowledge of different opinions with regards to sexuality and sexual orientation | 1 study; Hanass-Hancock et al., (2018) | Teachers |
|  | Knowledge of different opinions with regards to sexuality and religion | 1 study; Howard-Barr (2005) | Teachers |
|  | Tolerance of different opinions with regards to sexuality and sexual orientation | 1 study; Hanass-Hancock et al., (2018) | Teachers |

## Supplementary material 3: Figure 2 PRISMA Flow Diagram: study selection process on the Sexual Consent and Education Assessment (SCEA)


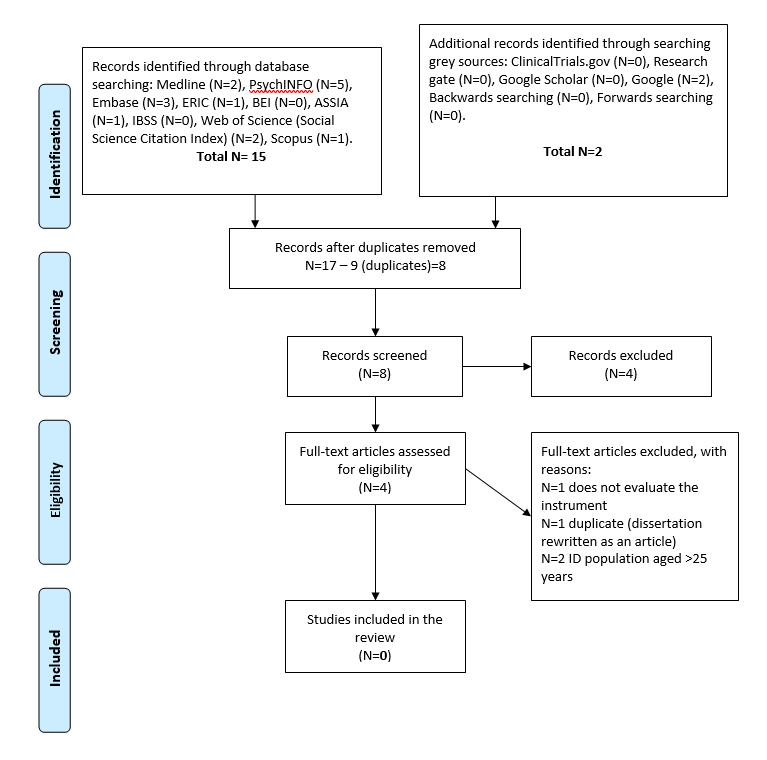


## Supplementary material 4: Figure 3 PRISMA Flow Diagram: study selection process on the Sexuality Knowledge, Experience, Feelings and Needs Scale for people with Intellectual Disability (Sex Ken-ID)


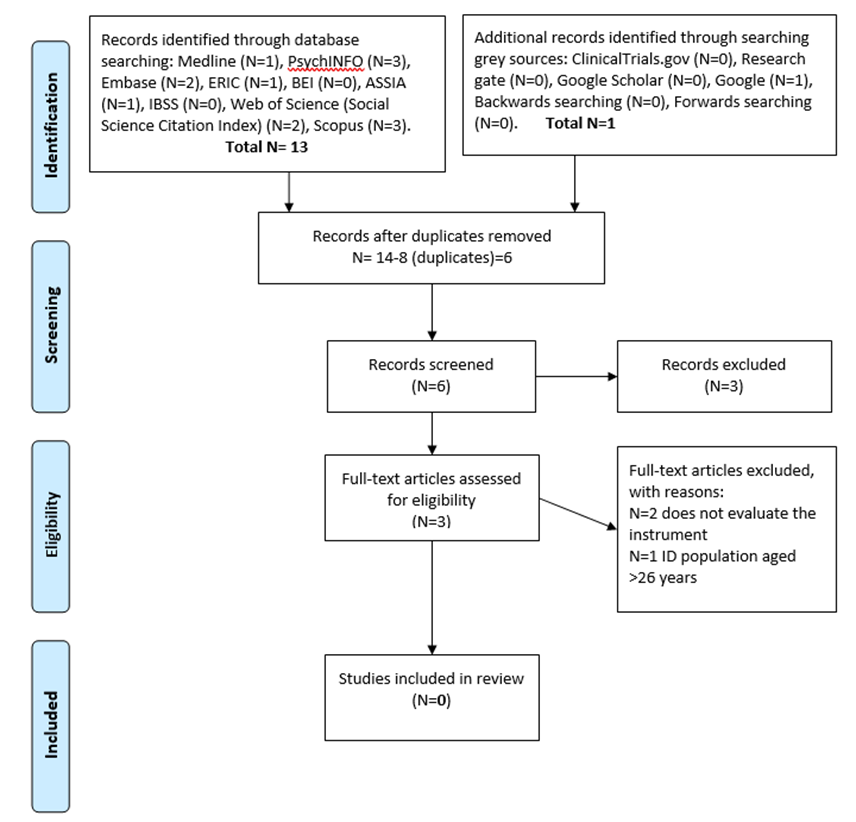


## Supplementary material 5: Figure 4 PRISMA Flow Diagram: study selection process on the Not a Child Anymore


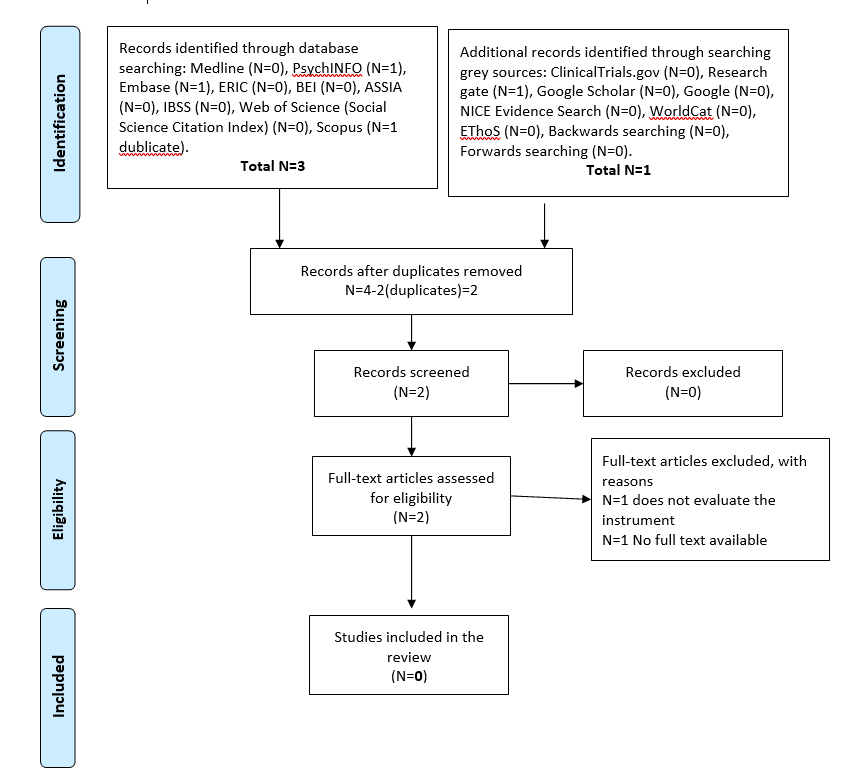

Supplement: Supplementary file 1 — Table S1: Example of search strategy used in the 1st and 2nd stage of review Table S2: RSE outcomes for students with intellectual disability that age was not specified Figure S1: PRISMA Flow Diagram: study selection process on the Sexual Consent and Education Assessment (SCEA) Figure S2: PRISMA Flow Diagram: study selection process on the Sexuality Knowledge, Experience, Feelings and Needs Scale for people with Intellectual Disability (Sex Ken‐ID) Figure S3: PRISMA Flow Diagram: study selection process on the Not a Child Anymore [file JIR-66-577-s001.docx]
